# Anticryptosporidial action mechanisms of *Launaea spinosa* extracts in *Cryptosporidium parvum* experimentally infected mice in relation to its UHPLC-MS metabolite profile and biochemometric tools

**DOI:** 10.1371/journal.pone.0317497

**Published:** 2025-03-03

**Authors:** Mai M. Elghonemy, Mohamed G. Sharaf El-Din, Dina Aboelsoued, Mohamed F. Abdelhameed, Mohamed A. El-Saied, Nagwa I. Toaleb, Mohamed A. Farag, Abdelsamed I. Elshamy, Abdelbaset M. Elgamal

**Affiliations:** 1 Department of Natural Compounds Chemistry, National Research Centre, Giza, Egypt; 2 Pharmacognosy Department, Faculty of Pharmacy, Port Said University, Port Said, Egypt; 3 Parasitology and Animal Diseases Department, Veterinary Research Institute, National Research Centre, Giza, Egypt; 4 Pharmacology Department, Medical Research and Clinical Studies Institute, National Research Centre, Giza, Egypt; 5 Department of Pathology, Faculty of Veterinary Medicine, Cairo University, Giza, Egypt; 6 Pharmacognosy Department, Faculty of Pharmacy, Cairo University, Cairo, Egypt; 7 Department of Chemistry of Microbial and Natural Products, National Research Centre, Giza, Egypt; University of British Columbia, CANADA

## Abstract

**Background:**

*Cryptosporidium parvum*, a leading cause of diarrhea, is responsible for millions of food and waterborne illnesses in humans and animals worldwide. *Launaea spinosa* (Asteraceae family) is a common herb found in the desert of the Mediterranean region, encompassing the peninsula of Sinai. Traditionally, it has been utilized for managing gastrointestinal issues and inflammation.

**Methods and findings:**

The present study aimed to assess *Launaea spinosa* (LS) extracts viz. ethyl acetate (LS-EtOAc), ethanol (LS-EtOH), and *n*-butanol (LS-BuOH), of different polarities against *C. parvum* in experimentally infected mice based on immunological, biochemical, histo- and immunohistochemical assays. Extracts were characterized via UHPLC-ESI-LIT-Orbitrap-MS and metabolite profiles were subjected to correlation modeling with bioactivities *via* supervised Partial Least Square (PLS) to identify active agents. Most *L. spinosa* extracts reduced fecal *C. parvum* oocyst count and mucosal burden (*P* < 0.05) than untreated infected mice, with LS-BuOH (200 mg/kg) exerting the highest reduction percentage (97%). These extracts increased immunoglobulin G (IgG) levels in infected and treated mice at all examined days post treatment. Also, the highest Interferon-Gamma (IFN-γ) and Interleukin-15 (IL-15) levels were obtained after 10 days of post inoculation (dPI), which were restored to a healthy state after 21 days, concurrent with a decrease in Tumor Necrosis Factor-Alpha (TNF-α) (*P* < 0.001). The increased liver enzyme (alanine aminotransferase, aspartate aminotransferase, and alkaline phosphatase) levels with infection were likewise reduced with extract administration. The LS extracts caused a significant increase in antioxidant glutathione peroxidase (GSH-Px) and catalase (*P < *0.001). Examination of colon tissue revealed that infected-treated mice with LS extracts exhibited a reduction in the expression of cleaved caspase-3, damage score, and degenerative changes. Metabolite profiling of different *L. spinosa* extracts led to the identification of 86 components, primarily phenolic acids, flavonoids, triterpenoid saponins, and fatty acids, with the first report of sulfated triterpenoid saponins in *Launaea* genus. PLS regression analysis revealed that bioeffects were significantly positioned close to LS-BuOH extract (R^2^: 0.9) mostly attributed to triterpenoid saponins and flavonoid glycosides.

**Conclusions:**

This study demonstrated potential anti-cryptosporidial effects of LS extracts, especially LS-BuOH, suggesting its potential for inclusion in future nutraceuticals aimed at *C. parvum* treatment.

## 1. Introduction

*Cryptosporidium* is a zoonotic intracellular protozoan parasite which is one of the major contributors to diarrhea in humans and animals [1,2]. It is considered the main cause of waterborne disease outbreaks worldwide [3] and is responsible for more than eight million foodborne disease cases annually worldwide [4]. In livestock animals, *Cryptosporidium* infection varies depending on animal species, geographical area, rearing practices, and the diagnostic tools used [5]. It is more frequent in neonate and young calves, with severity depending on age, calf immunity, dose of infection, geographical distribution, season, and mixed infection with other pathogens [6]. *Cryptosporidium parvum* (*C. parvum*) is widely spread in cattle herds causing significant economic losses, high morbidity, growth retardation, high treatment costs and representing a dominant cause of calf scours [5,7]. Currently, no prophylactic, therapeutic or natural products are fully effective against *C. parvum* infection either in humans or animals [8]. Consequently, there is increasing interest in identifying new therapeutics [9]. Many therapeutic agents have been tested for the treatment of cryptosporidiosis either *in vitro* or *in vivo,* albeit with limited effect [10]. Few have shown efficacy in reducing *C. parvum* oocyst shedding and reducing the severity and duration of diarrhea [11].

Numerous therapeutic and pharmacological uses of medicinal plants and their bioactive byproducts are well known. Across the world, *Launaea* Cass. (Family Asteraceae) is a common genus, particularly in Africa, the South Mediterranean, and Asia, with ca. 54 species [12]. *Launaea* plants exhibit traditional uses worldwide including the treatment of gastrointestinal illnesses, inflammation, wounds, fever, diarrhea, and infections of the stomach, breast, liver, skin, and infections of insects [12,13]. Biological assays have also verified the antibacterial, antioxidant, hypoglycemic, insecticide, cancer prevention, fungicide, anti-inflammatory, and anti-angiogenic capabilities of several *Launaea* species extracts and/or their isolated compounds [13–15]. *L. spinosa* (Forssk.) Sch. Bip. ex Kuntze is commonly found in the desert regions of Palestine, Jordan, Egypt (Sinai), and northwest Saudi Arabia [16]. It has traditionally been employed to treat fever, pain, wounds, skin diseases, gastrointestinal problems and inflammation [17]. The health advantages of L. spinosa are mediated through its phytochemical components such as flavonoids, coumarins, sesquiterpenes, and essential oils [12,18,19].

The current study was conducted with the following objectives: (i) assess the anti-parasitic action mechanism of *L. spinosa* extracts of varying polarities, ethyl acetate (EtOAc), hydro-ethanol (EtOH), and *n*-butanol (BuOH), against molecularly identified *Cryptosporidium parvum* in experimentally infected mice; (ii) identify bioactive metabolites in extracts of varying composition; and (iii) illustrate the relationship between bioactivities and extracts’ identified metabolites using biochemometric tools, such as partial least square (PLS).

## 2. Materials and methods

### 2.1. Plant material and extracts preparation

Aerial parts of *L. spinosa* were collected from Wadi Hagul, in the Egyptian Eastern Sahara (30°02′34.3° N, 32°05′40.6° E) during the flowering stage in May 2022. The Plant Ecology Professor at Mansoura University, Dr. Ahmed M. Abd-ElGawad, authenticated and verified the species and the voucher specimen, Mans. 001121905, was placed in the Faculty of Science` herbarium of Mansoura University.

Collected *L. spinosa* aerial parts were cleaned first from dirt and then left in an open, fully dry, and shady area for 21 days, until they were completely dry. The dried plant material was finely powdered using a clean plant grinder. Three equally weighed portions (500 g each) were randomly aliquoted from the 1.5 kg of powdered aerial dried parts of *L. spinosa* to be subjected for 5 days to the different extraction solvents. Ethyl acetate (EtOAc, 2L), 70% hydro-ethanol (EtOH, 2L), and *n*-butanol (*n*-BuOH, 2L) were used to extract each part separately. After filtration, the solvent was evaporated under reduced pressure using a rotavapor at 45–50 °C to yield three separated black residues weighing 17.8, 22.3, and 19.2 g, respectively. Extracts were kept in three opaque glass vials at a temperature of –4 °C until further assays or analysis.

### 2.2. Biological assaying

#### 2.2.1. Ethical approval.

All animal experimental procedures were performed at the National Research Centre (NRC) Animal House and Immunology and Parasitology Laboratory, Veterinary Research Institute, NRC. All methods and experiments were performed in accordance with the relevant guidelines and regulations of the International Animal Ethics Committee guidelines and the Institutional Guidelines of the National Research Centre Animal Research Committee (Approved Protocol No. 8444052023) and in accordance with ARRIVE guidelines.

#### 2.2.2. Isolation and identification of *Cryptosporidium* oocysts.

Fecal samples were collected from 20 diarrheic newborn buffalo calves (aged 10–20 days) reared by local farmers in Giza Governorate, Egypt, from the calves’ rectum using sterile latex gloves in separate clean labeled containers. The collected samples were transported to Immunology and Parasitology Lab, NRC, at the day of collection, then prepared and stained with modified Ziehl-Neelsen (MZN) staining [20] and Carbol fuchsin [21], and then examined under a light microscope (LEICA Imaging Systems Ltd., England) with oil immersion at the day of collection. *Cryptosporidium* oocysts were concentrated using Sheather’s sugar solution floatation method [22]. Oocysts were collected and stored at 4°C in a 2.5% potassium dichromate solution (K_2_Cr_2_O_7_, Sigma-Aldrich, Canada). Genomic DNA was extracted from 10 isolated oocyst samples using a DNA extraction Kit (GeneDireX Inc., USA) according to the manufacturer’s protocol, and DNA concentration was measured by a microvolume spectrophotometer (Q9000, Quawell, USA) and stored at − 20°C until further analysis. For *Cryptosporidium* sp. identification, amplification of the extracted DNA was performed using PCR targeting *Cryptosporidium* oocyst wall protein (COWP) gene, 553 bp, (Cry9: Forward 5′-GGACTGAAATACAGGCATTATCTTG-3′ and Cry15: Reverse 5′- GTAGATAATGGAAGAGATTGTG-3′) according to Feltus [23] using a thermal Cycler (BIO-RAD, Singapore). PCR products were visualized using Molecular Imager (BIO-RAD, Singapore) in 1.5% Agarose gel electrophoresis stained with RedSafe (Intron Biotechnology, Republic of Korea), and estimated for band size with a 100 bp ladder (QIAGEN, USA).

*Cryptosporidium*-positive PCR products were purified using Gel Extraction Kit (Qiagen, USA) following the manufacturer’s instructions. Purified products were sequenced with Big Dye Terminator V3.1 Cycle Sequencing Kit (Perkin-Elmer, USA) using an automated sequencer (ABI 3130, Applied Biosystems, USA). The resulting sequences were corrected by ChromasPro 1.7 software (Technelysium Pty Ltd., Australia) and then compared with those available in GenBank using nucleotide BLAST (https://blast.ncbi.nlm.nih.gov/Blast.cgi) and submitted in GenBank. Multiple sequence alignment was performed as designed by Thompson [24] using CLUSTAL W 1.83 of MegAlign module of Lasergene DNAStar software Pairwise. Phylogenetic analysis was performed with maximum likelihood, neighbor-joining and maximum parsimony in MEGA6 software [25].

#### 2.2.3. Antigen and hyperimmune serum assay.

Infective *C. parvum* oocysts (identified previously by PCR) were pooled from corresponding oocyst inoculums, washed 3 times with Phosphate Buffered Saline (PBS) solution (pH = 7.2), counted by a hemocytometer, and diluted in double distilled water to obtain infection dose 10^5^ oocysts/mL [26]. Ten parasite-free Swiss albino mice, 3 weeks old, obtained and housed in good conditions at specific cages at the Animal House, NRC, Egypt, with free access to food and water, were infected orally using gastric tubes 1 h before meal by 10^5^ *C. parvum* oocysts in 250 μL PBS solution (pH = 7.2). After four days, mice fecal pellets were collected daily for 3 weeks, and examined with MZN staining [20]. Oocysts in fecal pellets were isolated by Sheather’s sugar solution. *C. parvum* antigen was prepared from isolated oocysts according to Kaushik et al., 2009 [27] using Vibra Cell VCX750 Sonicator (Sonics & Materials, USA). Then, antigen protein content was estimated by Lowry’s method [28] and stored at –20 ºC until use.

Hyperimmune serum was prepared by immunization of 2 parasite-free rabbits, housed in good conditions at specific cages at the Animal House, NRC, Egypt, with free access to food and water, using 40 µg/Kg *C. parvum* antigen according to Fagbemi [29]. After immunization, blood samples were collected from the rabbits’ ear vein as described by Aboelsoued [30]. Serum was separated by centrifugation and stored at –20 ºC for further use.

#### 2.2.4. Affinity purification of *C. parvum* antigen.

The rabbit hyperimmune serum was defrosted and dialyzed for three days in a coupling buffer (0.1 M NaHCO_3_, 0.5 M NaCl, pH = 8.4) and then coupled to 2 mg/mL-swollen beads of cyanogen bromide-activated Sepharose-4B (CNBr Sepharose-4B, Sigma-Aldrich, USA). The prepared *C. parvum* antigen was applied to the affinity column (Flex-Column, Kimble, USA) as described by Aboelsoued et al. [8]. The protein content of the purified antigen was estimated as described by Lowry’s method [28] and then stored at –20 ºC for further use.

#### 2.2.5. Experimental design of the *L. spinosa* extracts` inhibitory effects against *C. parvum.*

Forty-five mice were randomly divided into nine groups (five mice/ each). **Group 1**: Control negative group (uninfected-untreated). **Group 2**: Control positive group, mice were infected orally using gastric tubes 1 h before meal by 1x10^5^ *C. parvum* oocysts in 250 μL PBS solution (pH = 7.2) [26]. **Group 3**: Mice were infected by *C. parvum* oocysts and treated with 100 mg Nitazoxanide as a reference drug. **Group 4**: Mice were infected by *C. parvum* oocysts and treated with 100 mg/Kg of *L. spinosa* ethyl acetate extract (LS-EtOAc). **Group 5**: Mice were infected by *C. parvum* oocysts and treated with 200 mg/Kg of LS-EtOAc. **Group 6**: Mice were infected by *C. parvum* oocysts and treated with 100 mg/Kg of *L. spinosa* ethanol extract (LS-EtOH). **Group 7**: Mice were infected by *C. parvum* oocysts and treated with 200 mg/Kg of LS-EtOH. **Group 8**: Mice were infected by *C. parvum* oocysts and treated with 100 mg/Kg of *L. spinosa* butanol extract (LS-BuOH). **Group 9**: Mice were infected by *C. parvum* oocysts and treated with 200 mg/Kg of LS-BuOH.

All animals from Group 3 through Group 9 received oral treatments as described above once per day for 3 days starting from the third day of inoculation (after oocysts appeared in mice feces). After inoculation, all mice were observed for three weeks. About 200 μL of blood was collected from the retro-orbital sinus of each mouse using a sterile hematocrit capillary tube along the inner corner of the eye twice per week from all groups. Sera were separated and stored at –20 ºC for further use. At the end of experiment, all mice were sacrificed gently and rapidly by cervical dislocation under anesthesia using intraperitoneal injection of Sodium pentobarbital at dose of 40 to 50 mg/Kg and then disposed according to the Safety and Health Committee of the National Research Centre (NRC). Small intestines were collected and rinsed free of intestinal content and then cut in two sequential fragments taken from the ileum; one fragment was used for histopathological and immunohistochemical examination, and the other was used for mucosal *C. parvum* oocysts and developmental stages count detection by MZN staining. Bedding was changed every day and fecal samples were collected daily starting from the third day post inoculation (dPI) until the end of the experiment.

#### 2.2.6. Fecal oocysts’ shedding.

Fecal pellets were collected daily from all mice groups for the determination of the number of *C. parvum* oocysts output counted from each group. Each fecal sample was smeared on a glass slide, stained according to MZN [20]. The number of oocysts were counted in 50 microscopic fields (100X objective) [31]. Fecal pellets were collected from the uninfected mice group parallel to the infected groups and examined to confirm their negativity during the experiment. Percent of reduction (PR), representing the decrease in oocysts’ count of treated mice groups than the infected-untreated mice group, was calculated according to **Farid** [32] using the following formula:


$$\text{Percent of reduction (PR)}=\frac{\text{No.of oocystsin infected untreatedgroup}-\text{No.of oocystsin infected treated group}}{\text{No.of oocysts in infected untreated roup}}\text{X}100$$

#### 2.2.7. Quantification of mucosal burden.

At the 10^th^ day post inoculation (peak of oocyst shedding in infected untreated mice), two mice were sacrificed from each infected group for quantification of mucosal burden. Small intestine fragments taken from the ileum were cleaned free of intestinal content and homogenized in 10 volumes of PBS solution. Ten microliters of homogenate were smeared onto a glass slide then stained according to MZN [20]. Oocysts and developmental stages were counted on the whole slide and results were expressed as oocysts’ number/ mg of tissue.

#### 2.2.8. C. parvum specific antibodies detection.

Specific *C. parvum* Immunoglobulin G (IgG) titer in infected mice serum after infection and treatments at different intervals (0-day, 5 days post treatment (dPT), 10 dPT and 15 dPT) was monitored using indirect ELISA from the purified fraction isolated from *C. parvum* oocyst antigen as previously described by Priest [33]. Optical densities (OD) of the developed color were recorded at 450 nm using automated microplate ELISA reader (ELx800UV, BioTek, USA). Concentrations of the used antigen, sera and conjugates’ dilutions were calculated by checkerboard titration. Cut-off value was estimated by mean OD values of negative sera +  3 standard deviation (SD).

#### 2.2.9. Cytokine levels.

Using Sandwich ELISA kits, IFN-γ, IL-15 (Sunlong Biotech, China), and TNF-α (Sigma-Aldrich, Saint Louis, USA) levels were assessed in mice sera as described by the manufacturer. Optical density was measured using automated microplate ELISA reader (ELx800UV, BioTek, USA) at 450 nm. Concentrations were estimated using standard curves performed at the same assays.

#### 2.2.10. Biochemical marker assays.

Liver enzymes including alanine aminotransferase (ALT), aspartate aminotransferase (AST), alkaline phosphatase (ALP), and antioxidant parameters: glutathione peroxidase (GSH-Px) and catalase were determined using colorimetric method according to manufacturer using kits (Biodiagnostic, Giza, Egypt). The colorimetric reaction was measured using UV-Visible spectrophotometer (Cary 100, Agilent Technologies, Inc., Australia).

#### 2.2.11. Histopathological examination of intestines from different experimental groups.

Intestinal specimens from each animal among experimental groups were collected and well-preserved in 10% neutral buffered formalin then routinely processed in alcohol followed by xylene and embedded in blocks of paraffin that sectioned at 5 µm in thickness, stained with hematoxylin and eosin (H&E) and examined under light microscope (Olympus BX43) connected to digital camera (Olympus DP27) with CellSens dimensions software (Olympus, Tokyo, Japan). The assessment of histopathological alternations was quantified in five random microscopical fields from each animal and scored from (0–3) as follows: (0) means no changes, (1) means mild change, (2) means moderate changes and (3) means severe changes. Concisely, the histopathological alterations were assigned for five parameters (mucosal apoptosis and necrosis, infiltration of inflammatory cells, congestion, mucosal edema, apoptosis, and necrosis of glandular epithelium). The total histopathological lesion score was obtained with summation of the five parameters evaluated altogether [34].

#### 2.2.12. Immunohistochemical expression of Cleaved Caspase-3.

The expression level of cleaved caspase-3 in the intestinal segment was analyzed using primary antibodies against caspase-3 (diluted 1:100; ab32042, Abcam) that were incubated overnight at 4˚C. The corresponding secondary antibody used was horseradish peroxidase (HRP)-labeled goat anti-mouse antibody (Abcam) by incubation for 2 h, after which diaminobenzidine tetrachloride (DAB, ThermoScientific) was used to visualize the immune reaction. The positive expression level was determined as a brown color that was assessed as area % using Olympus CellSens dimensions software (Olympus, Tokyo, Japan) [35].

### 2.3. Metabolites profiling of *L. spinosa* extracts *via* high-resolution ultra-performance liquid chromatography-mass spectrometry analysis (UHPLC-ESI-LIT-Orbitrap-MS)

The three *L. spinosa* extracts’ UHPLC-ESI-LIT-Orbitrap-MS analyses were performed under the same reported methodology and conditions in **Farrag** et al [36] and **Abdel Shakour** et al [37]. The analysis of the three *L. spinosa* extracts was conducted using both negative and positive modes of electrospray ionization (ESI) to achieve a comprehensive metabolome profile. The negative ionization mode demonstrated superior sensitivity, producing pronounced [M-H]^-^ ions and achieving higher signal-to-noise ratios with less background noise compared to the positive ionization mode. The identification of metabolites relied on several criteria: order of elution, ultraviolet-visible (UV/Vis) spectroscopy, molecular formulas predicted using high-resolution mass spectrometry (HR-MS), fragmentation patterns in tandem MS-MS, matching with known standards, and comparison against a proprietary database and Mass Bank (https://massbank.jp/) as well as the Dictionary of Natural Products (DNP, 2015).

### 2.4. Statistical analysis

Statistical analyses were performed with SPSS 19.0 for Windows (SPSS, Inc., USA) and values were given as Means ±  Standard Error. Data were tested for normality via Kolmogorov-Smirnov test of normality. A one-way analysis of variance (ANOVA) was conducted, followed by Tukey’s multiple comparisons test, to assess the statistical significance of variations among groups in terms of oocyst counts in fecal pellets and mucosal tissue, concentrations of ELISA, cytokines, and biochemical parameters. The non-parametric data of lesion score was compared by Kruskal-Wallis and Dunn’s multiple comparisons test. Significant differences were considered when *P* < 0.05.

## 3. Results

### 3.1. Biological results

#### 3.1.1. PCR and phylogenic analysis.

**Fig 1:**
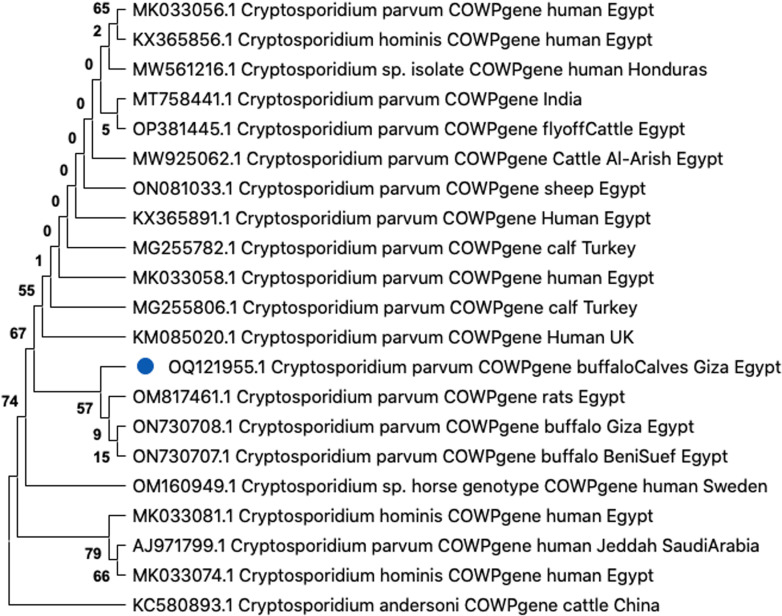
Phylogenetic tree based on the maximum likelihood method using *Cryptosporidium* oocyst wall protein (COWP) gene for *Cryptosporidium* spp. The obtained sequence is highlighted (blue dot).

DNA of *Cryptosporidium* sp. was detected in fecal samples of 10 out of 20 diarrheic newborn buffalo calves (50%) using the COWP gene. After sequencing and phylogenic analysis, *C. parvum* isolate (GenBank: OQ121955.1) was identified being 100% (495/495) identical to those of *C. parvum* detected in buffaloes from Egypt (GenBank: ON730708.1 and ON730707.1). Phylogenetic analysis revealed that this genotype was clustered in a well-supported branch with other *C. parvum* sequences (**Fig 1**).

#### 3.1.2. Fecal oocysts’ shedding.

**Fig 2:**
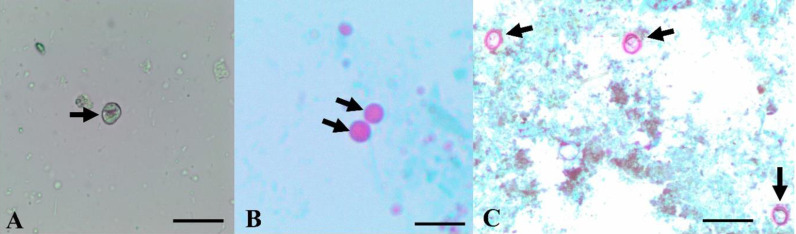
*C. parvum* oocysts in fecal pellets of infected untreated mice. A: Fresh fecal smear, and B: Modified Ziehl-Neelsen stained smear, 1000X). **C:** *C. parvum* oocysts in fecal pellets of infected mice which were treated with LS-BuOH extract showing oocyst shape deformation and lacking inner structures (1000X, Bar =  0.4µm).

Examination of mice fecal smears to detect *C. parvum* oocysts revealed that mice shed many oocysts from the 3^rd^ dPI and there was a gradual decrease in *C. parvum* oocysts’ shedding in the infected-untreated mice from the 10^th^ dPI which continued to decrease until no oocysts were detected in day 20 PI. In parallel, *C. parvum* oocyst counts in all infected and treated groups showed decline in LS-BuOH, LS-EtOH, NTZ, and LS-EtOAc from the 5^th^, 6^th^, 8^th^, 9^th^ dPI, respectively. There was a statistically significant difference (*P* < 0.05) between oocysts shedding in infected-untreated, *L. spinosa*-treated and NTZ-treated groups until reaching negligible oocyst counts or no oocysts. The LS-BuOH extract reduced *C. parvum* oocysts’ count significantly (*P* < 0.05) in experimentally infected mice than other *L. spinosa* extracts and NTZ treatments in almost all day’s PI (**Table 1**). Interestingly, *C. parvum* oocysts in fecal pellets of LS-BuOH treated mice appeared deformed in shape, lacking inner structure, as compared to infected-untreated and other treated mice fecal pellets (**Fig 2**).

**Table 1:** *C*.* parvum* oocysts’ shedding in feces of infected mice groups.

Mice Group dPI	Untreated	NTZ treated	*L. spinosa* extracts treated						F-Value
**Ethyl acetate** (LS-EtOAc)		**Ethanol** (LS-EtOH)		**Butanol** (LS-BuOH)		
**100mg/Kg**	**200mg/Kg**	**100mg/Kg**	**200mg/Kg**	**100mg/Kg**	**200mg/Kg**	
Day 3	132.67 ± 1.86	131.00 ± 1.53	132.00 ± 1.15	130.30 ± 2.3	128.67 ± 4.67	131.67 ± 1.3	132.67 ± 2.85	131.00 ± 3.06	0.26^NS^
Day 4	555.00 ± 1.53	555.67 ± 0.67	554.67 ± 2.73	554.00 ± 0.58	555.67 ± 0.88	553.33 ± 1.76	552.67 ± 1.7	554.60 ± 1.76	0.44^NS^
Day 5	637.00 ± 5.13^a^	542.60 ± 3.38^e^	582.67 ± 3.18^b^	544.67 ± 2.96^e^	580.00 ± 1.73^bc^	568.70 ± 2.6^cd^	584.30 ± 7.54^b^	558.30 ± 3.76^d^	52.4^*^
Day 6	679.6. ± 2.03^a^	582.00 ± 2^e^	658.00 ± 1.53^b^	609.30 ± 5.2^d^	645.00 ± 6.66^c^	616.00 ± 3.79^d^	380.67 ± 3.76^f^	328.00 ± 2.52^g^	1206.3^*^
Day 7	697.00 ± 2.52^a^	593.00 ± 3.5^d^	670.30 ± 2.4^b^	637.00 ± 3.61^c^	486.00 ± 5.29^e^	452.67 ± 5^f^	264.67 ± 7.3^g^	203.67 ± 4.18^h^	1701.6^*^
Day 8	714.00 ± 2.3^a^	650.30 ± 2^d^	686.30 ± 3.76^b^	667.00 ± 6.56^c^	365.00 ± 5.69^e^	307.30 ± 6.4^f^	103.30 ± 2^g^	74.00 ± 1.73^h^	3889.3^*^
Day 9	745.30 ± 2.6^a^	450.30 ± 3.76^d^	702.30 ± 3.38^b^	674.00 ± 17.5^c^	298.67 ± 1.45^e^	283.30 ± 2.96^e^	93.70 ± 2.9^f^	64.67 ± 0.88^g^	1643.4^*^
Day 10	784.30 ± 5.4^a^	426.67 ± 4.06^d^	647.30 ± 4.6^b^	611.00 ± 5.86^c^	171.00 ± 1.53^e^	120.30 ± 0.88^f^	61.67 ± 1.2^g^	23.70 ± 0.88^h^	6719.6^*^
Day 11	705.00 ± 3.2^a^	378.00 ± 5.29^d^	577.30 ± 3.76^b^	526.00 ± 2.65^c^	164.00 ± 1.15^e^	105.70 ± 3.18^f^	54.67 ± 0.88^g^	21.00 ± 1.15^h^	7674.7^*^
Day 12	683.30 ± 2.4^a^	222.00 ± 6.93^d^	499.30 ± 2.6^b^	444.70 ± 5.17^c^	121.67 ± 0.88^e^	93.30 ± 1.76^f^	42.00 ± 1.53^g^	12.30 ± 1.2^h^	5144.4^*^
Day 13	632.30 ± 3.38^a^	153.30 ± 7.13^d^	295.30 ± 2.9^b^	195.67 ± 4.37^c^	101.00 ± 0.58^e^	76.00 ± 4.16^f^	24.67 ± 1.86^g^	7.33 ± 1.2^h^	2946.4^*^
Day 14	602.67 ± 3.7^a^	95.00 ± 0.58^d^	166.00 ± 0.58^b^	121.70 ± 0.88^c^	85.70 ± 1.86^e^	57.00 ± 0.58^f^	24.00 ± 0.58^g^	5.00 ± 0.57^h^	15018.8^*^
Day 15	528.00 ± 3.79^a^	76.70 ± 0.88^d^	122.30 ± 1.2^b^	97.30 ± 0.88^c^	70.30 ± 0.88^e^	43.00 ± 0.57^f^	18.67 ± 0.33^g^	1.00 ± 0.58^h^	12202.8^*^
Day 16	495.00 ± 3.2^a^	55.00 ± 0.57^d^	111.30 ± 0.88^b^	84.67 ± 1.2^c^	43.70 ± 0.33^e^	31.00 ± 0.58^f^	14.00 ± 0.58^g^	0.67 ± 0.33^h^	15217.5^*^
Day 17	313.67 ± 3.28^a^	21.30 ± 1.2^e^	96.67 ± 0.88^b^	47.70 ± 1.45^c^	32.67 ± 0.33^d^	19.30 ± 0.33^e^	8.00 ± 0.58^f^	**0 ± 0** ^**g**^	5515.1^*^
Day 18	149.30 ± 3.48^a^	2.00 ± 2^e^	77.30 ± 0.88^b^	30.70 ± 1.45^c^	27.67 ± 0.33^c^	11.00 ± 0.58^d^	4.70 ± 0.33^e^	0 ± 0^e^	1091.8^*^
Day 19	55.00 ± 1.73^a^	**0 ± 0** ^**e**^	25.00 ± 1.73^b^	12.00 ± 1.15^c^	6.00 ± 1.7^d^	4.00 ± 1.2^de^	1.30 ± 0.88^e^	0 ± 0^e^	230.8^*^
Day 20	0 ± 0^c^	0 ± 0^c^	6.00 ± 0.58^a^	1.70 ± 0.667^b^	2.30 ± 0.33^b^	**0 ± 0** ^**c**^	**0 ± 0** ^**c**^	0 ± 0^c^	40.8^*^
Day 21	5 ± 2.65^a^	3.3 ± 0.88^ab^	1.67 ± 1.2 ^ab^	**0 ± 0** ^**b**^	**0 ± 0** ^**b**^	0 ± 0^b^	0 ± 0^b^	0 ± 0^b^	3.3^**^

Data is expressed as Mean ±  SE, Means followed by different letters indicate significance.

* Significant differences at *P <  0.001.*

** Significant differences at *P <  0.05.* dPI: days Post Inoculation, NTZ: Nitazoxanide.

**Fig 3:**
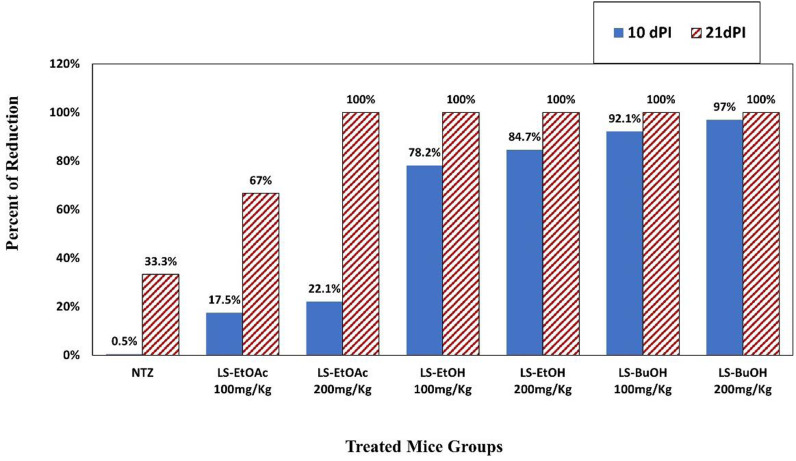
Percent of reduction in *C. parvum* oocysts’ shedding in feces of infected and treated mice groups compared with infected non-treated mice group. dPI: days Post Inoculation, LS-EtOAc: *L. spinosa* ethyl acetate extract, LS-EtOH: *Launaea spinosa* Ethanol extract, LS-BuOH: *Launaea spinosa *Butanol extract..

Regarding oocysts’ shedding reduction after 10 days of infection in the infected and treated mice groups, LS-BuOH extract 200 mg/Kg showed the strongest reduction (97%) between the experimental groups (Fig 3). At the 21^st^ dPI, almost all groups reached 100% oocyst reduction except in the case of NTZ and LS-EtOAc extract 100 mg/Kg treated groups (**Fig 3**).

#### 3.1.3. Mucosal burden.

Oocysts and developmental stages enumerated in mucosal tissue of infected-untreated mice exhibited the highest number after 10- and 21-dPI. Both concentrations of LS-BuOH extract (200 mg/Kg and 100 mg/Kg) showed the strongest significant anti-cryptosporidial effect (*P* < 0.05) on *C. parvum* oocysts’ mucosal burden (**Table 2**), and in agreement with fecal oocysts’ shedding PR shown in **Fig 3**.

**Table 2:** *C*.* parvum* oocysts and developmental stages count in intestine of infected mice groups.

Mice Group Count/ dPI		Untreated	NTZ treated	*L. spinosa* extracts treatment						F-Value
**Ethyl acetate** (LS-EtOAc)		**Ethanol** (LS-EtOH)		**Butanol** (LS-BuOH)		
**100mg/Kg**	**200mg/Kg**	**100mg/Kg**	**200mg/Kg**	**100mg/Kg**	**200mg/Kg**	
Oocysts & Developmental Stages	**10 Days**	1020 ± 5.2^a^	833.67 ± 4.63^b^	807.67 ± 5.93^c^	801 ± 6.66^c^	684.33 ± 7.54^d^	657.67 ± 2.9^e^	95 ± 4.51^f^	75.67 ± 2.33^g^	4442.59^*^
	**21 Days**	18 ± 1.53^a^	6 ± 1.73^b^	9 ± 1.53^b^	4.67 ± 0.88^bc^	1.33 ± 1^cd^	0.33 ± 0.3^d^	0 ± 0^d^	0 ± 0^d^	10.74^*^

Data is expressed as Mean ±  SE, Means followed by different letters indicate significance.

* Significant differences at *P <  0.001.* dPI: days Post Inoculation, NTZ: Nitazoxanide.

#### 3.1.4. The humoral immune response of healthy, infected, and treated mice.

**Fig 4:**
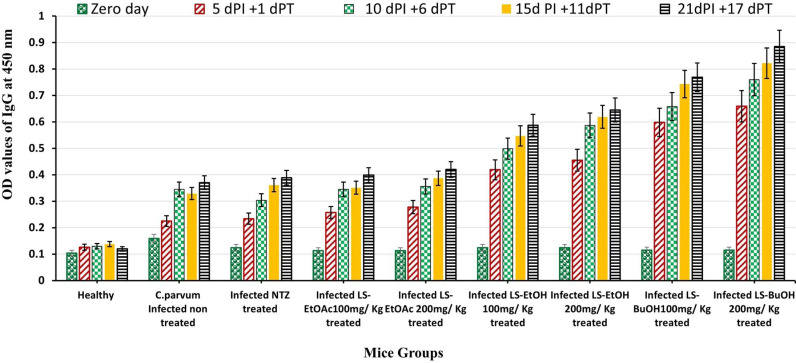
Serum IgG level in healthy and *C. parvum* infected mice untreated and treated groups at zero, 5, 10, 15 and 21-dPI. dPI: days Post Inoculation, dPT: days Post Treatment, *C. parvum: *Cryptosporidium* parvum*, LS-EtOAc: *Launaea spinosa* Ethyl acetate extract, LS-EtOH: *Launaea spinosa* Ethanol extract, LS-BuOH: *Launaea spinosa* Butanol extract.

Levels of specific *C. parvum* antibodies IgG showed a significant increase in the infected and treated mice (*P < 0.05*) at all dPT as follows: 1 dPT (5^th^ dPI), 6 dPT (10^th^ dPI), 11dPT (15^th^ dPI), 17 dPT (21^st^ dPI), compared to infected-untreated mice. IgG levels increased gradually from the first dPT with highest level detected at 17 dPT in mice from group 5 treated with 100 and 200 mg/Kg LS-BuOH extract at an OD value of 0.885 and 0.769, respectively, as compared with other *L. spinosa* extracts treated groups (**Fig 4**).

#### 3.1.5. Cytokines profile.

The levels of serum cytokines including IFN-γ, IL-15 and TNF-α were significantly (*P* <  0.001) higher in serum of *C. parvum* experimentally infected-untreated mice group after 10 and 21 dPI compared to the control healthy rats (**Table 3**). Treatment of mice by Nitazoxanide and *L. spinosa* ethyl acetate and ethanol extracts showed significantly (*P* <  0.001) lower IFN-γ levels compared to the infected-untreated mice.

**Table 3:** Serum INF-γ, IL-15 and TNF-α levels in healthy and *C. parvum* infected mice with different treatments.

Mice Group Cytokine/ dPI		Uninfected untreated	Infected								F-Value
**Infected untreated**	**NTZ treated**	***L. spinosa* extracts treatment**						
		**Ethyl acetate** (LS-EtOAc)		**Ethanol** (LS-EtOH)		**Butanol** (LS-BuOH)		
		**100mg/Kg**	**200mg/Kg**	**100mg/Kg**	**200mg/Kg**	**100mg/Kg**	**200mg/Kg**	
**IFN-γ** **(ng/mL)**	**10 Days**	78.43 ± 0.33^h^	130.3 ± 0.58^d^	126.7 ± 0.67^e^	117.4 ± 0.56^g^	123.5 ± 0.^f^	124.4 ± 0.42^ef^	134.8 ± 0.4^c^	195.92 ± 1.8^b^	218.2 ± 1.5^a^	298.16^*^
	**21 Days**	78.8 ± 0.8^g^	165.74 ± 0.94^a^	161.3 ± 3.02^b^	145.09 ± 0.67^c^	132.68 ± 0.5^e^	139.35 ± 0.7^d^	137.78 ± 0.74^d^	95.09 ± 0.67^f^	79.07 ± 0.9^g^	521.54^*^
**IL-15** **(ng/mL)**	**10 Days**	153.19 ± 0.9^f^	180.6 ± 1.99^e^	203.2 ± 1.95^d^	206.39 ± 1^cd^	214.6 ± 6.04^bc^	212.3 ± 1.98^bcd^	219.18 ± 6.9^b^	233.3 ± 0.61^a^	239.73 ± 1.1^a^	2285.6^*^
	**21 Days**	151.37 ± 0.8^h^	218.04 ± 2.25^a^	201.37 ± 1.97^c^	214.6 ± 0.8^a^	206.16 ± 1.1^b^	193.38 ± 1.2^d^	179 ± 0.99^e^	163.7 ± 0.79^f^	158.67 ± 1.6^g^	731.41^*^
**TNF-α** **(ng/mL)**	**10 Days**	84.25 ± 1.44^f^	210.92 ± 5.07^a^	122.17 ± 1.1^d^	146.33 ± 1.1^b^	141.75 ± 1.4^bc^	136.75 ± 1.4^c^	120.92 ± 0.8^d^	116.75 ± 1.4^d^	105.5 ± 0.7^e^	63.61^*^
	**21 Days**	82.58 ± 0.83^f^	306.75 ± 7.64^a^	128.42 ± 2.2^bc^	134.08 ± 1.3^b^	121.75 ± 1.4^cd^	124.25 ± 1.44^c^	113.83 ± 1.1^d^	92.58 ± 2.2^e^	83.42 ± 2.2^f^	332.47^*^

Data is expressed as Mean ±  SE, Means followed by different letters indicate significance.

* Significant differences at *P <  0.001.* dPI: days Post Inoculation, NTZ: Nitazoxanide, IFN-γ: Interferon-Gamma, IL-15: Interleukin-15, TNF-α: Tumor Necrosis Factor-Alpha.

In contrast, the LS-BuOH extract group showed significantly (*P* <  0.001) higher levels of IFN-γ after 10 dPI at a dose of 100 mg/Kg (195.92 ±  1.77 ng/mL) and reached its maximal level at 200 mg/Kg (218.2 ±  1.5 ng/mL) compared to other infected groups. After 21 dPI, IFN-γ levels decreased in the serum of the LS-BuOH extract group at both doses towards a healthy state, and there was no significant difference between the LS-BuOH extract (200 mg/Kg) group and the healthy uninfected group. In contrast, IL-15 level was significantly (*P* <  0.001) elevated in *L. spinosa* extract groups after 10 dPI compared to infected-untreated group, reaching highest levels at the concentration of 200 mg/Kg (239.73 ± 1.1ng/mL). After 21 dPI, IL-15 levels were significantly (*P* <  0.001) lower than those of infected-untreated mice, and their levels were lowest at the concentration of 200 mg/Kg (158.67 ± 1.6 ng/mL).

Regarding TNF-α, there was a concentration-dependent decrease (*P* < 0.001) in its level in infected mice treated with *L. spinosa* extracts. *L. spinosa* butanol extract (200 mg/Kg) was determined to be superior to other *L. spinosa* extracts as there was no significant differences in TNF-α levels between *L. spinosa* butanol extract (200 mg/Kg) and the healthy uninfected-untreated group (**Table 3**).

#### 3.1.6. Liver functions and antioxidant assays.

Concerning levels of liver enzymes, there was a significant (*P < *0.001) elevation in ALT, AST, and ALP levels in *C. parvum* experimentally infected-untreated mice (93.26 ±  0.94, 263.5 ±  1.4, and 293.5 ±  1.4 U/L, respectively) compared to healthy uninfected-untreated ones (28.89 ±  0.82, 127.03 ±  2.3, and 203.7 ±  4.58 U/L, respectively) after 21 dPI (17 dPT). Liver enzyme levels were significantly (*P < *0.001) lower in mice treated with Nitazoxanide or *L. spinosa* extracts compared with the infected-untreated group. Among different *L. spinosa* extracts, the greatest effect was observed in butanol extract at a dose of 200 mg/Kg as manifested by no significant differences in ALT, AST and ALP levels between this treatment and healthy group (Table 4).

**Table 4:** Serum liver function parameters (ALT, AST and ALP) and antioxidant activity parameters (GSH-Px and Catalase) in healthy and *C. parvum* infected mice with different treatments at day 21 PI.

Mice Group Parameter	Uninfected untreated	Infected								F-Value
**Infected untreated**	**NTZ treated**	***L. spinosa* extracts treatments**						
		**Ethyl acetate** (LS-EtOAc)		**Ethanol** (LS-EtOH)		**Butanol** (LS-BuOH)		
		**100mg/Kg**	**200mg/Kg**	**100mg/Kg**	**200mg/Kg**	**100mg/Kg**	**200mg/Kg**	
**ALT (U/L)**	28.89 ± 0.82^g^	93.26 ± 0.94^a^	72.26 ± 1.39^c^	73.95 ± 0.68^bc^	62.7 ± 1.09^d^	76.58 ± 1.27^b^	58.36 ± 0.48^e^	46.6 ± 1.05^f^	31.26 ± 0.85^g^	473.01^*^
**AST (U/L)**	127.03 ± 2.3^e^	263.5 ± 1.4^a^	193.36 ± 3^b^	193.76 ± 2.3^b^	183.5 ± 1.4^c^	176 ± 2.59^c^	161.19 ± 1.1^d^	155.97 ± 1.6^d^	132.37 ± 4.83^e^	258.59^*^
**ALP (U/L)**	203.7 ± 4.58^e^	293.5 ± 1.4^a^	254.36 ± 1.05^c^	268.43 ± 3^b^	263.5 ± 1.4^b^	262.67 ± 1.4^b^	254.52 ± 2.2^c^	237.97 ± 2.3^d^	209.03 ± 3.25^e^	127.79^*^
**GSH-Px (U/mL)**	9.09 ± 0.24 ^a^	5.19 ± 0.05^c^	6.95 ± 0.25^b^	5.82 ± 0.35^c^	6.98 ± 0.58^b^	5.98 ± 0.09^c^	6.79 ± 0.09^b^	7.38 ± 0.12^b^	8.98 ± 0.17^a^	25.091^*^
**Catalase (U/mL)**	279.84 ± 0.56^a^	210.46 ± 3.4^e^	233.18 ± 2.6^cd^	221.26 ± 5.5^de^	235.3 ± 10^cd^	233.93 ± 6^cd^	253.19 ± 1.1^b^	246.3 ± 2.7^bc^	275.06 ± 2.2^a^	23.817^*^

Data is expressed as Mean ±  SE, Means followed by different letters indicate significance.

* Significant differences at *P <  0.001.* PI: Post Inoculation, NTZ: Nitazoxanide, ALT: Alanine aminotransferase, AST: Aspartate aminotransferase, ALP: Alkaline phosphatase, GSH-Px: Glutathione peroxidase.

As shown in Table 4, GSH–Px and catalase serum activities decreased significantly (*P < *0.001) in *C. parvum* infected-untreated mice compared to healthy mice after 21 dPI. In contrast, GSH–Px and catalase were significantly (*P < *0.001) higher in mice treated with Nitazoxanide or *L. spinosa* extracts compared to the infected-untreated group. Once again, *L. spinosa* butanol extract (200 mg/Kg) was determined to be superior to other *L. spinosa* extracts as levels were almost equal to those detected in the healthy mice group (**Table 4**).

#### 3.1.7. Histopathological finding.

**Fig 5:**
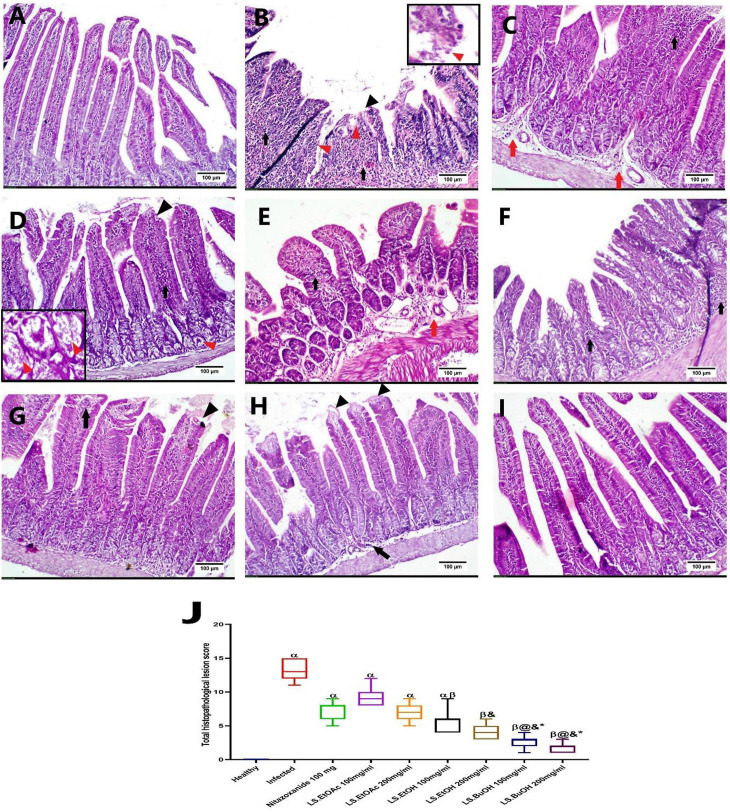
Representative photomicrographs showed H & E-stained intestinal sections. A) Group 1: Healthy group, showing negative expression, B) Group 2: *C. parvum* infected untreated group, C) Group 3: Infected and treated with Nitazoxanide 100mg/Kg treated group, D) Group 4: Infected and treated with *L. spinosa* ethyl acetate extract (LS-EtOAc) 100mg/Kg., E) Group 5: Infected and treated with *Ls*. EtOAc 200mg/Kg, F) Group 6: Infected and treated with *L. spinosa* ethanol extract (LS-EtOH) 100mg/Kg. G) Group 7: Infected and treated with LS-EtOH 200mg/Kg. H) Group 8: Infected and treated with *L. spinosa* butanol extract (LS-BuOH) 100mg/Kg. I) Group 9 Infected and treated with LS-BuOH 200mg. Note: inflammatory cells (black arrow), edema (red arrow), *C. parvum* oocyst (red arrowhead), and sloughing and necrosis of villous epithelium (black arrowhead). J) Box-plot diagram representing the total histopathological lesion score among experimental groups; α: significant difference from the healthy group, β: significant difference from the infected group, @: significant difference from Nitazoxanide 100mg/Kg group, &: significant difference from *Ls.* EtOAc 100mg/Kg group, * : significant difference from *Ls.* EtOAc 200mg/Kg group. A significant difference was considered when *P* < 0.05.

Microscopically, normal intestinal architecture (mucosa, crypt, submucosa, muscularis layer and serosa) was detected in the healthy mice group. In contrast, the intestinal section of infected groups showed diffuse lymphoplasmacytic cells infiltrated in the lamina propria-submucosa in addition to sloughing and necrosis of lamina epithelialis, congestion of blood vessels and edema with presence of different developmental stages of *C. parvum* in the intestinal epithelium and crypt of Lieberkühn that suffered from degenerative changes (**Fig 5**). Meanwhile, the infected-treated mice with Nitazoxanide 100 mg/Kg revealed multi-focal aggregation of inflammatory cells in lamina propria with edema. The intestine of the infected-treated mice with LS-EtOAc 100 mg/Kg exhibited sloughing and necrosis of the villous tip with congestion and edema beside the presence of *C. parvum* oocysts in intestinal crypts, causing degeneration and necrosis of intestinal crypt. On the other hand, marked edema and congestion with focal aggregation of inflammatory cells was detected in the infected-treated mice with LS-EtOAc 200 mg/Kg. Noticeable improvement was observed in the infected-treated mice with LS-EtOH 100 and 200 mg/Kg, respectively. Both groups showed mild to moderate sub-epithelial inflammatory cell infiltration, edema and sloughing of some villous lining epithelium. Marked amelioration of the intestinal histopathological alterations was detected in the infected-treated mice with LS-BuOH 200 mg/Kg within both doses as they showed the lowest lesion score compared with other treated groups. The examined intestinal sections of butanol extract likewise revealed a reduction in the injury score (**Fig 5**).

Regarding the histopathological lesion score, there was a statistically significant difference (*P* < 0.001) between healthy and other experimental groups except for the infected-treated mice with LS-EtOH at the dose 200 mg/Kg, and the infected-treated mice with LS-BuOH at both doses 100 mg/Kg and 200 mg/Kg. No statistically significant difference (*P* > 0.05) was detected between infected mice, the infected-treated mice with Nitazoxanide 100 mg/Kg, or the infected-treated mice with LS-EtOAc 100 mg/Kg and 200 mg/Kg. Meanwhile, the infected-treated mice with LS-EtOH 200 mg/Kg showed a significant decline (*P* < 0.001) in estimated lesion score compared to the 100 mg/Kg LS-EtOH group. Both doses of LS-BuOH (100 and 200 mg/Kg) revealed no significant difference (*P* > 0.05) as both showed the weakest intestinal lesions. Different intestinal injuries are illustrated in **Fig 5**.

#### 3.1.8. Immunohistochemical assay of cleaved caspase-3 expression.

**Fig 6:**
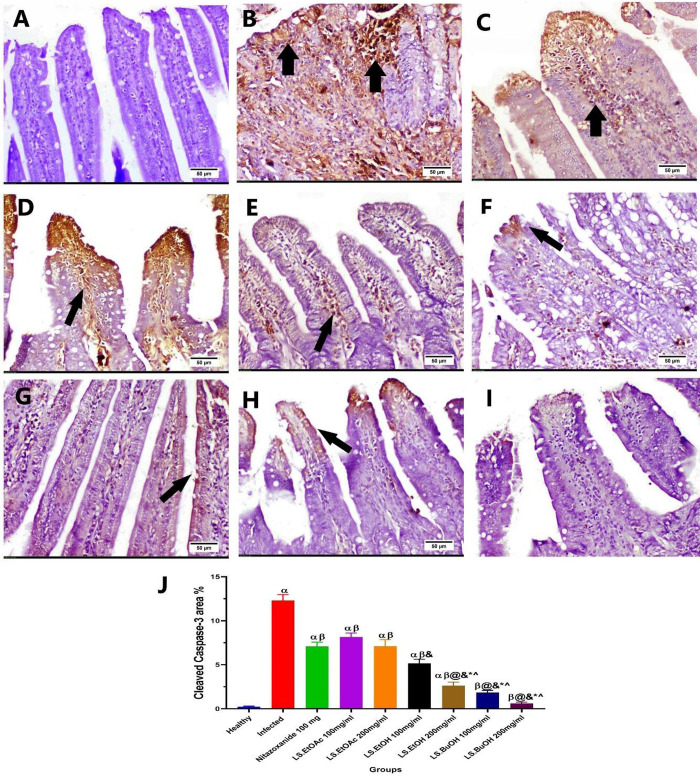
Representative photomicrographs of cleaved Caspase‑3 immune‑stained intestinal section. A) Group 1: Healthy group, showing negative expression, B) Group 2: *C. parvum* infected untreated group, C) Group 3: Infected and treated with Nitazoxanide 100mg/Kg treated group, D) Group 4: Infected and treated with *L. spinosa* ethyl acetate extract (LS-EtOAc) 100mg/Kg., E) Group 5: Infected and treated with *Ls*. EtOAc 200mg/Kg, F) Group 6: Infected and treated with *L. spinosa* ethanol extract (LS-EtOH) 100mg/Kg. G) Group 7: Infected and treated with LS-EtOH 200mg/Kg. H) Group 8: Infected and treated with *L. spinosa* butanol extract (LS-BuOH) 100mg/Kg. I) Group 9 Infected and treated with LS-BuOH 200mg/. Note: black arrow indicates positive expression. J) Chart representing the cleaved Caspase-3 area percent among experimental groups; α: significant difference from the healthy group, β: significant difference from the infected group, @: significant difference from Nitazoxanide 100mg/Kg group, &: significant difference from LS-EtOAc 100mg/Kg, * : significant difference from LS-EtOAc 200mg/Kg, ^ significant difference from LS-EtOH 100mg/Kg. Data presented as mean ± standard deviation (SD). A Significant difference was considered when *P* <  0.05.

Negative expression of cleaved caspase-3 was detected in the intestinal segment of the healthy untreated mice group (G1) (**Fig 6A**). In contrast, intense positive expression of caspase-3 was determined in the intestinal section of the *C. parvum* infected-untreated mice group (G2) (**Fig 6B**). Meanwhile, expression declined significantly (*P* < 0.05) in all treated groups compared to the infected group. Moderate immunoreactivity was detected in the infected-treated mice with Nitazoxanide 100 mg/Kg (**Fig 6C**), the infected-treated mice with LS-EtOAc 100 mg/Kg (**Fig 6D**), and 200 mg/Kg (Fig 6 E), respectively, though no statistically significant differences were observed. Otherwise, the expression decreased significantly (*P* < 0.001) in the infected-treated mice with LS-EtOH 100 mg/Kg (**Fig 6F**) and LS-EtOH 200 mg/Kg (**Fig 6G**). Conversely, weak immunoreactivity was observed in both butanol extract groups (the infected-treated mice with LS-BuOH 100 mg/Kg (**Fig 6H**) and LS-BuOH 200 mg/Kg (**Fig 6I**)) with no statistically significant difference between them. Expression levels of cleaved caspase-3 protein are presented in **Fig 6J**.

### 3.2. UHPLC-ESI-LIT-Orbitrap-MS-based metabolites profiling of *Launaea spinosa* extracts

**Fig 7:**
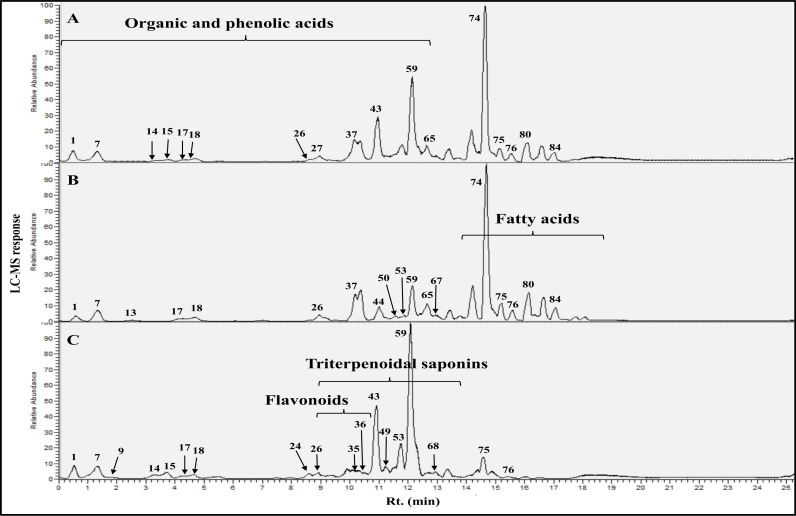
Representative UPLC-ESI-MS negative ionization mode spectra of the different *L. spinosa* extracts. (**A**) ethyl acetate, (**B**) ethanol, and (**C**) butanol. Assigned peak numbers follow that shown in **Table 5**.

**Fig 8:**
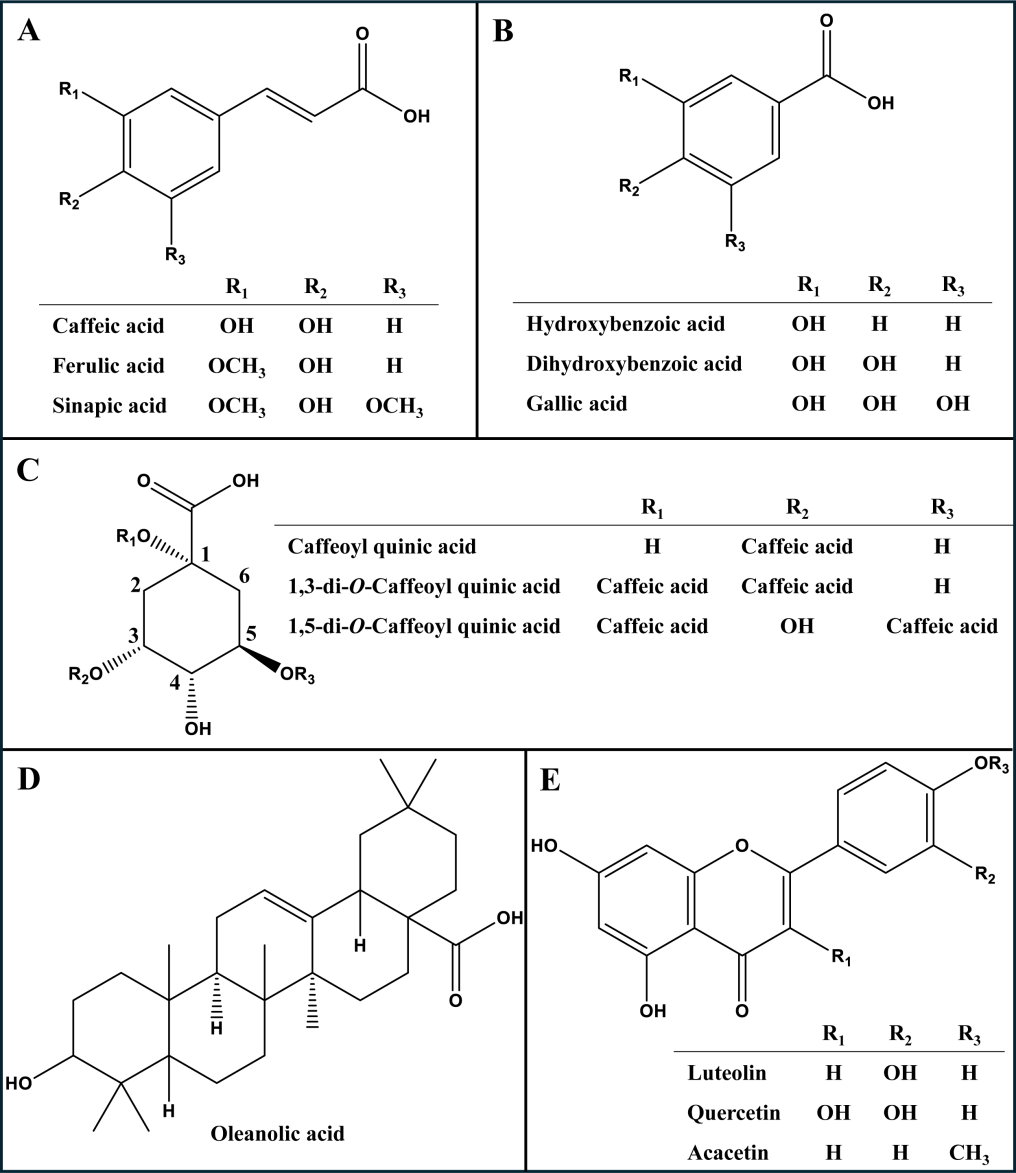
Major classes of metabolites. Hydroxycinnamates (**A**), hydroxybenzoates (**B**), quinic acid conjugates (**C**), triterpenoid based oleanolic acid (**D**), and flavonoids (**E**), identified in *L. spinosa* extracts with selected compound(s) discussed in the text.

Reversed-phase UHPLC-ESI-LIT-Orbitrap-MS analysis was employed to holistically compare metabolite profiles of *L. spinosa* extracts *viz*. ethanol, ethyl acetate, and butanol, and to identify markers for each fraction to likely mediate for anti-parasitic effects observed in an untargeted manner. Both negative and positive ion modes were conducted to annotate 86 metabolites belonging to different classes, comprising alcohols, fatty acids, flavonoids, organic acids, phenolic acids, triterpenes, and triterpenoid saponins, as shown in **Table 5**. UHPLC-ESI-LIT-Orbitrap-MS negative mode chromatograms of *L. spinosa* extracts and structures of the main identified metabolic classes are shown in **Fig 7** and **8**, respectively. Metabolites were eluted based on their polarity, starting with the most polar compounds such as organic acids, phenolic acids, and alcohols, followed by compounds of moderate polarity, including flavonoids and triterpenoid saponins, with the least polar metabolites, fatty acids, being eluted last in the chromatogram as detailed for each metabolite class in the next subsections.

**Table 5:** Metabolites identified in the different *L. spinosa* extracts using UHPLC-ESI-MS in negative/ positive ionization modes.

No.	Rt (min)	UV	Metabolite	[M-H]^-^/[M+ H] ^+^ (m/z)	Error (ppm)	Elemental composition	MS^n^ product ions	*L. spinosa* extract		
**EtOAc**	**EtOH**	**BuOH**
1	0.51	191, 295	Quinic acid	191.0565	1.8	C_7_H_11_O_6_^-^		+	**+**	**+**
2	0.58	n.d.	Malic acid	133.0147	2.5	C_4_H_5_O_5_^-^	115, 87, 71	+	**+**	**+**
3	0.63	n.d.	Citric acid	191.0200	3.5	C_6_H_7_O_7_^-^	173, 111	+	**+**	**+**
4	0.70	n.d.	Succinic acid	117.0197	2.5	C_4_H_5_O_4_^-^	99, 73	+	**+**	**+**
5	1.10	264, 321	Protocatechuic acid-*O*-hexoside	315.0721	0.0	C_13_H_15_O_9_^-^		+	**+**	**+**
6	1.18	264, 310	Piscidic acid	255.0512	0.8	C_11_H_11_O_7_^-^	237, 211, 193, 179, 165, 149	+	**+**	**+**
7	1.29	277, 310	Ferulic acid	193.0509	1.5	C_10_H_9_O_4_^-^		+	**+**	**+**
8	1.60	259, 289	Dihydroxybenzoic acid	153.0196	1.3	C_7_H_5_O_4_^-^	135, 123, 109, 91	+	**+**	**–**
9	1.87	268	Syringic acid-*O*-hexosie	359.0983	‒0.1	C_15_H_19_O_10_^-^	197, 153	+	**+**	**+**
10	2.05	277, 327	*O*-Caffeoylquinic acid	353.0876	‒0.6	C_16_H_17_O_9_^-^	191, 179, 173, 161, 135	+	**+**	**+**
11	2.54	236, 295	Hydroxybenzoic acid	137.0248	2.6	C_7_H_5_O_3_^-^	119, 110, 93, 66	+	**+**	**+**
12	2.68	259, 289	Dihydroxybenzoic acid isomer	153.0196	1.3	C_7_H_5_O_4_^-^	135, 123, 109, 91	+	**–**	**–**
13	2.82	n.d.	Hydroxyheptanedioic acid	175.0615	1.5	C_7_H_12_O_5_^-^	157, 147, 131, 115, 113, 85	+	**–**	**–**
14	3.17	259	Coniferyl alcohol sulfate	259.0282	0.6	C_10_H_11_O_6_S^-^	229, 179, 161	+	**+**	**+**
15	3.72	259	Coniferyl alcohol sulfate Isomer	259.0283	0.5	C_10_H_11_O_6_S^-^	229, 179, 161	+	**+**	**+**
16	4.02	289	Eucomic acid	239.0562	0.5	C_11_H_11_O_6_^-^	221, 177, 163, 149, 133, 91	+	**+**	**+**
17	4.17	304, 327	*O*-Caffeoylquinic acid isomer1	353.0877	‒0.4	C_16_H_17_O_9_^-^	191, 179, 173, 161, 135	+	**+**	**+**
18	4.64	299, 325	*O*-Caffeoylquinic acid isomer2	353.0876	‒0.6	C_16_H_17_O_9_^‒^	191, 179, 173, 161, 135	+	**+**	**+**
19	4.95	251, 327	Caffeic acid	179.0351	0.7	C_9_H_7_O_4_^‒^	161, 151, 135, 107	+	**–**	^+^
20	4.97	260, 316	Ferulic acid sulfate	273.0074	‒0.1	C_10_H_9_O_7_S^‒^	229, 193, 149, 79	+	**+**	**+**
21	5.95	290, 326	*O*-Caffeoylquinic acid isomer3	353.0876	‒0.7	C_16_H_17_O_9_^‒^	191, 179, 173, 161, 137, 135, 111	+	**+**	**+**
22	7.36	n.d.	Dihydroxy-(methylpropyl)butanedioic acid-*O*-rhamnoside	351.1297	0.0	C_14_H_23_O_10_^‒^	333, 315, 291, 267, 249, 231, 205, 175, 157, 113	+	**+**	**+**
23	8.45	293, 325	Methyl jasmonate-*O*-hexoside	431.1920	‒0.6	C_20_H_31_O_10_^‒^	385, 223, 205, 179, 153	+	**+**	**+**
24	8.57	291, 323	Hydroxyferuloyl-*O*-hexoside	371.0982	-0.4	C_16_H_19_O_10_^‒^	353, 249, 231, 209, 193, 175, 157, 121	+	**+**	**+**
25	8.71	297, 313	Sinapoyl-hexoside	385.1170	7.8	C_17_H_21_O_10_^‒^	325, 263, 241, 223, 181, 151, 121	+	**–**	^+^
26	8.81	294, 327	Di-*O*-caffeoylquinic acid	515.1193	‒0.5	C_25_H_23_O_12_^‒^	353, 191, 179, 173	+	**+**	**+**
27	8.97	295, 321	Caffeoylquinic acid methyl ether	367.1032	2.2	C_17_H_19_O_9_^‒^	191, 173	+	**+**	**+**
28	9.11	219, 334	Coniferin sulfate	421.0810	8.0	C_16_H_21_O_11_S^‒^	241, 179	+	**+**	**+**
29	9.16	n.d.	Octanedioic acid	173.0822	1.3	C_8_H_13_O_4_^‒^	111	+	**+**	**–**
30	9.48	261, 309	Hydroxybenzoic acid isomer	137.0247	2.1	C_7_H_5_O_3_^‒^	93	+	**+**	**–**
31	9.55	295, 330	Caftaric acid-*O*-hexoside	473.0723	‒0.6	C_22_H_17_O_12_^‒^	311, 293	+	**–**	**–**
32	9.56	335	*O*-Galloyl-*O*-(4-hydroxybenzoyl)- hexoside	451.0915	7.2	C_20_H_19_O_12_^‒^	435, 371, 311, 257, 241, 177	–	**–**	^+^
33	9.73	288, 369	Quercetin-*O*-rhamnosyl-hexoside	609.1458	‒0.5	C_27_H_29_O_16_^‒^	463, 447, 301, 271, 255	+	**+**	**+**
34	9.76	288, 369	Quercetin-*O*-hexoside	463.0881	‒0.3	C_21_H_19_O_12_^‒^	301	+	**–**	^+^
35	10.03	270, 330	Luteolin-*O*-rhamnosyl-hexoside	593.1509	‒0.4	C_27_H_29_O_15_^‒^	447, 285, 257, 213, 185, 171	+	**+**	**+**
36	10.10	294, 335	Tetrahydroxymethoxyflavone- *O*-rhamnosyl-hexoside	623.1617	-0.1	C_28_H_21_O_16_^-^	477, 447, 315, 299, 271, 255	+	**+**	**+**
37	10.12	295, 325	Di-*O*-caffeoylquinic acid isomer 1	515.1194	‒0.2	C_25_H_23_O_12_^‒^	353, 335, 191, 179	+	**+**	**+**
38	10.27	n.d.	Nonanedioic acid (Azelaic acid)	187.0978	1.4	C_9_H_15_O_4_^‒^	169, 125, 97	+	**+**	**–**
39	10.53	286, 321	Tetrahydroxyflavane-*O*-hexoside	435.0966	7.5	C_20_H_19_O_11_^‒^	343, 311, 249, 273, 241, 151	+	**+**	**+**
40	10.62	290, 323	*O*-Caffeoylferuloylquinic acid	529.1350	0.2	C_26_H_25_O_12_^-^	367, 353, 349, 335, 191, 179, 161	+	**+**	**–**
41	10.68	296, 324	Acacetin	285.0764	2.4	C_16_H_13_O_5_^ + ^		+	**–**	**–**
42	10.72	297, 325	Di-*O*-caffeoylquinic acid isomer 2	515.0497	5.8	C_25_H_23_O_12_^‒^	497, 435, 395, 377, 353, 335, 255, 191, 179	+	**+**	**+**
43	10.93	n.d.	Dihydroxy-oxo-oleanenoic acid-*O*-dihexoside sulfate	889.3892	3.2	C_42_H_65_O_18_S^‒^	845, 727, 683, 647, 603, 441, 401, 255	+	**+**	**+**
44	11.06	n.d.	Dihydroxy-oleanenoic acid-*O*-dihexoside	809.4326	‒0.7	C_42_H_65_O_15_^‒^	765, 647, 603, 585, 455, 391	+	**+**	**+**
45	11.10	289, 321	Tri-*O*-caffeoylquinic acid	677.1506	‒0.8	C_34_H_29_O_15_^‒^	515, 497, 353, 335	+	**+**	**+**
46	11.14	n.d.	Dihydroxy-oxo-oleanenoic acid-*O*-dihexoside sulfate isomer	889.3892	3.2	C_42_H_65_O_18_S^‒^	845, 727, 683, 603, 441, 401, 255	–	**+**	**+**
47	11.18	n.d.	Dihydroxy-oleanenoic acid-*O*- dihexosyl-pentoside sulfate	1007.4519	‒0.8	C_47_H_75_O_21_S^‒^	977, 845, 683, 653, 603, 471	–	**+**	**+**
48	11.18	n.d.	Dihydroxy-oxo-oleanenoic acid-*O*-dihexosyl-rhamnoside	955.4912	0.4	C_48_H_75_O_19_^‒^	833, 793, 749, 731, 705, 629, 587, 569, 455, 441	+	**–**	**–**
49	11.22	n.d.	Unknown triterpenoid saponin	859.3789	0.1	C_41_H_63_O_17_S^-^	815, 697, 653, 573, 483, 441	+	**+**	**+**
50	11.44	n.d.	Dihydroxy-oxo-oleanenoic acid-*O*-hexoside	931.3999	3.8	C_47_H_63_O_19_^‒^	887, 769, 725, 683, 441, 423, 283	+	**+**	**+**
51	11.51	n.d.	Dihydroxy-oxo-oleanenoic acid-*O*-hexosyl-rhamnoside sulfate	873.3945	0.2	C_42_H_65_O_17_S^‒^	829, 711, 667, 649, 587, 441, 401, 297	+	**+**	**+**
52	11.57	n.d.	Oleanene-diol-*O*-malonyl- dihexoside	851.4434	0.0	C_44_H_67_O_16_^-^	833, 807, 747, 689, 645, 603, 483, 441	+	**–**	**–**
53	11.69	n.d.	Unknown triterpenoid saponin	845.3993	4.1	C_44_H_61_O_16_^-^	815, 683, 653, 471, 441	+	**+**	**+**
54	11.86	n.d.	Tetrahydroxy-octadecenoic acid	345.2284	0.5	C_18_H_33_O_6_^-^	327, 309, 291	+	**+**	**–**
55	11.90	n.d.	Dihydroxy-oxo-oleanenoic acid-*O*-hexoside	647.3803	0.3	C_36_H_45_O_10_^-^	603, 565, 525, 513, 483, 455, 441	+	**+**	**–**
56	11.98	n.d.	Hydroxy-oleanenoic acid-*O*-dihexosyl-hexouronide	955.4906	‒0.2	C_48_H_75_O_19_^-^	793, 631, 455, 437	+	**+**	**+**
57	11.99	n.d.	Dihydroxy-oxo-oleanenoic acid-*O*-hexoside sulfate	727.3370	0.4	C_36_H_55_O_13_S^-^	683, 603, 563, 485, 441, 423, 259	+	**+**	**–**
58	12.01	n.d.	Trihydroxy-octadecenoic acid	329.2334	3.6	C_18_H_33_O_5_^‒^	293, 229, 211, 171	+	**+**	**–**
59	12.03	n.d.	Unknown triterpenoid saponin	1019.4526	‒2.6	C_48_H_75_O_21_S^‒^	857, 737, 607, 575, 510, 401, 357, 321	+	**+**	**+**
60	12.15	n.d.	Dihydroxy-oxo-oleanenoic acid-*O*-hexoside sulfate	727.3367	‒0.2	C_36_H_55_O_13_S^‒^	683, 667, 637, 603, 593, 596, 483, 441, 423, 370	+	**+**	**–**
61	12.26	n.d.	Oleanene-diol-*O*-hexoside	603.3904	0.2	C_35_H_55_O_8_^-^	441, 423, 391, 347, 327	+	**+**	**–**
62	12.36	n.d.	Hydroxy-oleanenoic acid-*O*-hexosyl-hexouronide	793.4374	-0.8	C_42_H_65_O_14_^-^	631, 613, 569, 455	+	**+**	**+**
63	12.42	n.d.	Hydroxy-oleanenoic acid-*O*-hexoside sulfate	697.3261	4.5	C_35_H_54_O_12_S^-^	653, 635, 617, 573, 485, 455, 441, 411	+	**+**	**+**
64	12.48	n.d.	Unkown triterpenoid saponin	1019.4517	‒1.0	C_48_H_75_O_21_S^‒^	939, 857, 401, 357, 321	–	**–**	^+^
65	12.52	n.d.	Trihydroxy-octadecadienoic acid	327.2180	0.9	C_18_H_31_O_5_^-^	309, 291, 247	+	**+**	**+**
66	12.80	n.d.	Dihydroxy-oleanenoic acid-*O*-pentoside-sulfate	683.3467	5.3	C_35_H_55_O_11_S^-^	639, 603, 638, 471, 210	+	**+**	**+**
67	12.84	n.d.	Trihydroxy octadecatrienoic acid	325.2022	2.2	C_18_H_29_O_5_^-^	307, 289, 281, 271, 263, 151, 137, 125	–	**+**	**+**
68	12.87	n.d.	Hydroxy-oleanenoic acid-*O*-hexosyl-pentoside-sulfate	829.4045	‒0.5	C_41_H_65_O_15_S^‒^	667, 587, 455, 373	+	**+**	**+**
69	13.22	n.d.	Hydroxy-oleanenoic acid-*O*-hexosyl-hexouronide isomer	793.4374	‒0.7	C_42_H_65_O_14_^‒^	749, 731, 631, 613, 587, 569, 537, 523, 513, 455, 437	+	**+**	**+**
70	13.45	n.d.	Dihydroxy-oleanenone	441.3371	0.0	C_29_H_45_O_3_^-^	399, 373, 361, 343, 325, 307	+	**+**	**+**
71	13.51	n.d.	Unknown triterpenoid saponin	1075.5139	1.8	C_55_H_79_O_21_^‒^	1045, 913, 883, 767, 669, 627, 455, 401	–	**–**	^+^
72	13.79	n.d.	Dihydroxy-octadecenoic acid	313.2384	‒0.1	C_18_H_33_O_4_^‒^	295, 277, 201, 171	+	**+**	**+**
73	14.17	n.d.	Dihydroxy-hexadecenoic acid	285.2074	1.0	C_16_H_29_O_4_^-^	267, 223	+	**+**	**+**
74	14.58	n.d.	Dihydroxy-octadecadienoic acid	311.2230	0.6	C_18_H_31_O_4_^-^	293, 249	+	**+**	**+**
75	15.09	n.d.	Dihydroxy-octadecenoic acid	313.2387	0.1	C_18_H_33_O_4_^-^	295, 251	+	**+**	**+**
76	15.41	n.d.	Unknown diterpene	339.2542	3.4	C_20_H_35_O_4_^-^	321, 295, 277, 253, 183	+	**+**	**+**
77	15.49	n.d.	Glycerol-dihydroxyeicosenoate	415.3065	‒0.1	C_23_H_43_O_6_^‒^	397, 377, 341, 323, 279, 241, 191	+	**+**	**+**
78	15.58	n.d.	Dihydroxy-nonadecenoic acid	327.2544	4.2	C_19_H_35_O_4_^-^	309, 255	+	**+**	**–**
79	15.84	n.d.	Hydroxyoctadecadienoic acid	295.2281	4.5	C_18_H_31_O_3_^-^	277, 249, 231, 193, 155, 141	+	**+**	**–**
80	15.97	n.d.	Dihydroxy-eicosenoic acid	341.2697	‒0.2	C_20_H_37_O_4_^‒^	323, 279	+	**+**	**+**
81	16.15	n.d.	Unknown triterpene	517.2805	‒0.3	C_29_H_41_O_8_^‒^	473, 441, 297, 219, 175	+	**+**	**+**
82	16.24	n.d.	Hydroxyhexadecanoic acid	271.2281	0.7	C_16_H_31_O_3_^‒^	253, 225, 209	+	**+**	**–**
83	16.36	n.d.	Acetylglycerol-dihydroxyeicosenoate	457.3171	2.4	C_25_H_45_O_7_^-^	441, 415, 397, 395, 369, 341, 323, 279	+	**+**	**–**
84	16.91	n.d.	Eicosanedioic acid	369.3010	‒0.1	C_22_H_41_O_4_^‒^	351, 307	+	**+**	**+**
85	17.40	n.d.	Hydroxydocosanoic acid	355.3216	‒0.4	C_22_H_43_O_3_^‒^	337, 309, 295	+	**+**	**–**
86	17.89	n.d.	Dihydroxytetracosenoic acid	397.3323	2.8	C_24_H_45_O_4_^‒^	379, 353, 335	+	**–**	**–**

(+) and (‒) indicate presence and absence of a metabolite, respectively; n.d., not detected; EtOH, ethanol extract; EtOAc, ethyl acetate extract; BuOH, butanol extract.

#### 3.2.1. Identification of phenolics.

Phenolic acids are commonly identified in phytochemical studies as precursors for numerous phenolic metabolites. They are detected either as free or conjugated with sugars and various organic acids [38]. Owing to their significant polarity, phenolic acids typically appear earlier in chromatographic analyses, primarily under negative ion mode, which aligns with their acidic nature. Twenty-three phenolic acids were identified in *L. spinosa* extracts, 16 of which (**7**, **10**, **17**–**21**, **24**–**27**, **31**, **37**, **40**, **42 & 45**) belonged to hydroxycinnamic acid derivatives, whereas the remaining 7 peaks (**5**, **8**, **9**, **11**, **12**, **30** & **32**) belonged to hydroxybenzoates, **Table 5**. The identified cinnamates were detected as either free or conjugated with sugars or organic acids. Caffeoylquinic acid conjugates were the main identified ones with mono-, di-, or tri-*O*- caffeoyl moieties. Peaks **10**, **17**, **18**, and **21** at *m/z* 353.0876 C_16_H_17_O_9_^-^ exhibited UV spectra typical of caffeoylquinic acid, alongside two characteristic fragments at *m/z* 179 and 191 for caffeic and quinic acids, respectively, and were annotated as positional isomers of caffeoyl quinic acid, as shown in **Fig.** S1 in **S1 File**. Similarly, peaks **26**, **37**, and **42** showed similar mass spectra at *m/z* 515.1193 C_25_H_23_O_12_^‒^ with extra loss of 162 amu equivalent to a caffeoyl moiety and were annotated as di-*O*-caffeoylquinic acid positional isomers as shown in **Fig.** S2 in **S1 File**. Likewise, peak **45** at *m/z* 677.1506 C_34_H_29_O_15_^‒^ showed characteristic fragments at 515 and 353 amu due to successive loss of caffeoyl moieties, and were annotated as tri-*O*-caffeoylquinic acid as shown in **Table 5** [39].

Compared to cinnamate derivatives, derivatives of hydroxybenzoic acid displayed a distinctive fragment at *m/z* 153 and 135 corresponding to dihydroxy and hydroxybenzoic acids, respectively [38]. Peaks **8** and **12** [M-H]^‒^ at *m/z* 153.0196 C_7_H_5_O_4_^‒^ were annotated as dihydroxybenzoic acid positional isomers. Their mass spectrum showed characteristic fragments at *m/z* 135, 109, and 91 due to loss of [M-H_2_O]^‒^, [M-CO_2_]^‒^, and [M-CO_2_-H_2_O]^‒^, respectively (**Fig.** S3 in **S1 File**). It should be noted that hydroxybenzoates were detected at trace levels in comparison to hydroxycinnamates, inferring that *L. spinosa* biosynthesis is activated towards cinnamates production, and in accordance with that previously identified in *L. mucronata*. [13]

This study represents the first *L. spinosa* phenolic acids profile, noting that their levels were higher in butanol extract than other extracts, which likely accounted for its significant biological activity compared to other extracts.

#### 3.2.2. Identification of organic acids.

Eleven organic acids (**1**–**4**, **6**, **13**, **16**, **22–23**, **29** & **38**) were detected early in the chromatogram owing to their high polarity. Their annotation was based on MS^2^ fragments of ‒18 and ‒44 amu due to the loss of H_2_O molecules and carboxyl group, respectively [40]. For example, peak **2** at *m/z* 133.0170 C_4_H_5_O_5_^‒^ showed such a fragmentation pattern and was annotated as malic acid (**Fig.** S4 in **S1 File**). The identified organic acids were previously detected in *Launaea mucronate* ethanolic extract [13], while this is the first report of an organic acids profile in *L. spinosa*.

#### 3.2.3. Identification of flavonoids.

Six chromatographic peaks (**33–36**, **39** & **41**) were identified as flavonoids and their conjugates based on their UV spectra (200**–**600 nm). The identified flavonoids belonged to flavones (**35**, **36** & **41**), flavonol (**33 & 34**), and flavane (**39**) as shown in **Table 5**. Flavones exhibited UV max at 270 nm (Band II) and 335**–**350 nm (Band I). For example, peak **35** at *m/z* 593.1509 C_27_H_29_O_15_^‒^ showed two characteristic losses of ‒146 and ‒162 amu equivalent to rhamnosyl and hexosyl moieties, respectively, and were annotated as luteolin-*O*-rhamnosyl-hexoside as shown in **Fig.** S5 in **S1 File**. [41].

Flavonols exhibited UV max at 250**–**275 nm (Band II) and 352**–**380 nm (Band I). For example, peak **33** at *m/z* 609.14581 C_27_H_29_O_16_^‒^ showed similar ‒146 and ‒162 amu losses yielding quercetin aglycone and were annotated as quercetin-*O*-rhamnosyl-hexoside as shown in **Fig.** S6 in **S1 File** [42].

It is important to highlight that flavonoids were identified at trace levels, which suggests that major secondary metabolites in *L. spinosa* included triterpenoid saponins and phenolic acids as shown in **Table 5**. Flavonoid concentration was significantly higher in butanol extract in comparison to ethyl acetate and ethanol owing to their increased polarity.

#### 3.2.4. Identification of triterpenoid saponins.

Twenty-one triterpenoid saponins of oleanane skeleton were detected in different extracts of *L. spinosa*, particularly in the butanol extract owing to their improved recovery in that solvent (**Table 5**) and suggestive that butanol is the richest in that class. The major identified saponins aglycones were hydroxy-oleanenoic acid (*m/z* 455) in peaks **56**, **62**, **63**, **68**, and **69**, dihydroxy-oleanenoic acid (*m/z* 472) in peaks **44**, **47**, and **61**, dihydroxy-oxo-oleanenoic acid (*m/z* 486) in peaks **43**, **46**, **48**, **50**, **51**, **53**, **55**, **57**, and **60**, and **66**, oleanene-diol (*m/z* 442) [38]. The triterpenoidal type saponins were assigned based on the loss of ‒44 amu for carboxylic acid group, with detection predominantly occurring in negative ionization mode. The identification of glycosides type was determined from the neutral losses of ‒86, ‒132, ‒146, ‒162, and ‒176 corresponding to malonyl, pentose, deoxyhexose, hexose, and hexuronic acid, respectively [43]. For example, peak **56** at *m/z* 955.4906 C_48_H_75_O_19_^‒^ showed three successive losses of ‒162, ‒162, and ‒176 amu equivalent to two hexosyl units and hexuronic acid, respectively. This fragmentation yielded a triterpenoid aglycone at *m/z* 455 equivalent to hydroxy-oleanenoic acid, assigned as hydroxy-oleanenoic acid-*O*-dihexosyl-hexouronide (**Fig**. S7 in **S1 File**).

Nine sulfated triterpenoidal saponins (**43**, **46**, **47**, **51**, **57**, **60**, **63**, **66**, and **68**) were detected for the first time in *L. spinosa*. Sulfate esters were identified in negative mode by their distinctive neutral loss of ‒80 amu [44]. For instance, peaks **47** and **68** at *m/z* 1007.4519 C_47_H_75_O_21_S^‒^ and *m/z* 829.4045 C_41_H_65_O_15_S^‒^ exhibited the fragmentation pattern of triterpenoidal saponins. Additionally, a characteristic loss of sulfate ester was detected, assigned as dihydroxy-oleanenoic acid-*O*-di-hexosyl-pentoside sulfate and hydroxy-oleanenoic acid-*O*-hexosyl-pentoside-sulfate (**Fig.** S8 in **S1 File** and S9 in **S1 File**, respectively). Despite the ability to identify many triterpenoidal saponins, it was impossible to provide a clear structure assignment due to the inability to determine the precise location of functional groups.

It’s noteworthy that triterpenoid saponins exhibited the highest relative levels in the butanol extract, which likely accounts for its superior biological action in comparison to other extracts, *i.e.*, ethyl acetate and ethanol. This is the first report of the presence of sulfated triterpenoid saponins in genus *Launaea* and should be pursued in other species for conclusive note of its distribution pattern.

#### 3.2.5. Identification of fatty acids.

In the later elution region of the chromatogram, 15 fatty acid peaks were eluted due to their nonpolar nature, and for the same reason, their amounts were higher in ethyl acetate extract (**Table 5**). These fatty acids belonged to unsaturated, mono-, and polyunsaturated fatty acids. The identified unsaturated fatty acids were oxygenated, *i.e.*, hydroxy (**79**), dihydroxy (**72–75**, **78**, **79**, and **86**), trihydroxy (**58**, **65**, and **67**), or tetrahydroxy (**54**) fatty acids as shown in **Table 5**. Hydroxy fatty acids were annotated based on the loss of extra water molecule(s) in negative ionization mode. For example, peak **67** annotated as trihydroxy octadecatrienoic acid showed a molecular mass at *m/z* 325.2022 C_18_H_29_O_5_^‒^ and three characteristic fragment ions at *m/z* 307, 289, and 271 due to three successive losses of H_2_O, alongside another key fragment at *m/z* 281 due to the loss of CO_2_ (**Fig.** S10 in **S1 File**). Hydroxy fatty acids have previously been attributed for anti-inflammatory and anticancer effects [45]. Compared to an abundance of unsaturated fatty acids, only three saturated fatty acids, *i.e.*, peaks **82**, **84**, and **85,** were detected as hydroxyhexadecanoic acid, eicosanedioic acid, and hydroxydocosanoic acid, respectively (**Table 5**).

### 3.3. Correlation between bioactivities and major metabolites in *L. spinosa* extracts

**Fig 9:**
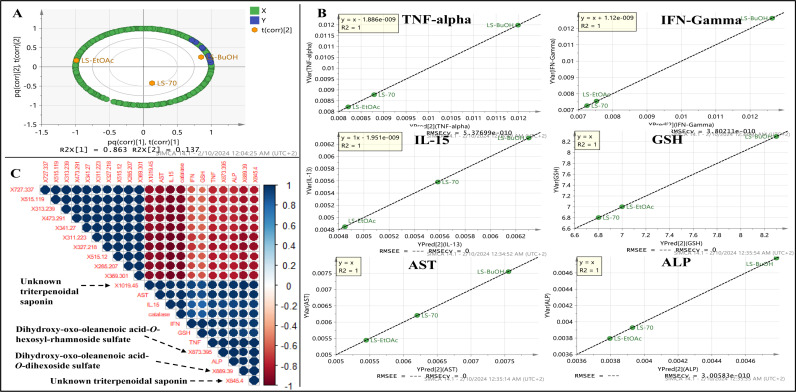
Partial Least Square (PLS) biplot. (**A**): PLS biplot of *L. spinosa* different extracts based on quantified metabolites by UPLC-MS negative mode designating metabolites’ level as (X-variables) correlated with investigated parameters (Y-variables) related to biological assay results, (**B**): Observed versus predicted effect for biological activity parameters, i.e., TNF-alpha, IFN-Gamma, IL-15, GSH, AST, and ALP, showing correlation in relation to peaks abundance detected in negative mode. The t(corr) [2] sign denotes the correlation sites between X- and Y-variables. (**C**) Correlogram visualizing correlation between metabolites analyzed using UPLC-MS in negative mode and antiparasitic activities. The color and size of the circles are proportional to the correlation coefficients. Positive correlations are shown in blue (different shades; dark blue with the strongest correlation) whereas negative correlations in red (ranging from light red to red; dark red with the weakest correlation). (For interpretation of the references to color in this figure legend, the reader is referred to the Web version of this article).

Supervised partial least square (PLS) was utilized to assess the relationship between identified metabolites *via* UPLC-MS (X-variables) and the anti-parasitic activities conducted in the present study. Partial Least Square biplot (**Fig. 9A**), represented by score and loading plots, revealed a clear distinction between the three different *L. spinosa* extracts and variables contributing to their separations (R2X = 86%). Interestingly, the biological activity parameters *viz*. TNF- α, IFN-Gamma, IL-15, GSH, AST, and ALP, (Y-variables) were positioned closely to the butanol extract on the plot, indicating a strong correlation with this extract (**Fig. 9A**), and in agreement with biological activity parameters revealing that LS-BuOH extract exhibited the strongest activity versus LS-EtOAc and LS-EtOH extracts.

To pinpoint the MS variables strongly linked to this differentiation, metabolites with a VIP score of 5 or higher were chosen to create a correlogram, aiming to elucidate their role in biological activities. The models’ validation was demonstrated by regression analysis, displaying an R2 value of 1 for all assessed parameters (**Fig 9B**). The correlogram (**Fig 9C**) indicated robust correlation among triterpenoid saponins, *i.e.*, dihydroxy-oxo-oleanenoic acid-*O*-dihexoside sulfate (peak **43**), dihydroxy-oxo-oleanenoic acid-*O*-hexosyl-rhamnoside sulfate (peak **51**), and two unknown triterpenoid saponins (peaks **53** and **59**), with correlation coefficients (R2) equal to or greater than 0.9. These correlations were observed in relation to all assessed antiparasitic activities, and infer that triterpenoidal saponins from *L. spinosa* mostly mediate for the measured bioactivities. Conversely, di-*O*-caffeoylquinic acid and hydroxylated fatty acids showed a negative correlation with the assessed antiparasitic activity parameters. There is a growing interest in triterpenoidal saponins, including their antioxidant, hepatoprotective, antiviral, and anticancer effects, as evidenced by recent studies [46–49]. This study revealed that *L. spinosa* butanol extract stands out as the most potent antiparasitic extract, primarily attributed to its rich triterpenoid saponins levels. Therefore, *L. spinosa* butanol extract may be considered for inclusion in nutraceuticals for parasite management.

## 4. Discussion

The control of cryptosporidiosis is mostly difficult because of large numbers of oocysts excreted in the infected host feces, contaminating the surrounding environment and acting as a source of infection for humans and animals [50]. In the present study, *Cryptosporidium* spp. was isolated from diarrheic newborn buffalo calves, and the species was confirmed as *C. parvum* by PCR*.* This isolate was previously detected in humans and animals worldwide [51] and the human disease resembles that found in neonatal calves [52], whichis considered the most pathogenic and dominant species in farm animals [53,54]. The *C. parvum* isolate was identical to those detected in buffaloes (GenBank: ON730708.1 and ON730707.1) and cattle (GenBank: MW925062.1 and MW925061.1) from Egypt.

In the current study, the mice were used to evaluate the anti-cryptosporidial effect of three prepared *L. spinosa* extracts on *C. parvum* via assessing fecal oocyst shedding, mucosal burden, humoral and cellular immune responses in mice sera, and histopathological and immunohistochemical changes in mice intestine.

In the present study, examined mice fecal pellets revealed that mice shed many *C. parvum* oocysts from the 3^rd^ dPI and there was a gradual decrease in oocysts’ shedding in the infected-untreated mice from the 10^th^ dPI (peak of oocyst shedding). This ensured that the infection was propagated in mice models with the selected oocysts’ inoculation dose, and this coincides with previous studies which evaluated the pattern of *C. parvum* oocysts’ shedding [55,56]. Herein, LS-BuOH extract reduced *C. parvum* oocysts’ count significantly (*P* < 0.05) in experimentally infected mice compared to other treated groups (97% at the 10^th^ dPI), the control group and untreated-infected mice. Similar findings were recorded in many studies using *in vitro* and *in vivo* animal models to demonstrate the effect of some plant extracts in treating diarrhea and enteritis caused by *Cryptosporidium* sp. such as: moringa [26], pomegranate peel [55], black seed [57], garlic [58,59], curcumin [60], and ginger [61]. In this study, *C. parvum* oocysts detected in infected mice were morphologically similar to other *C. parvum* shown in previous studies [26,62]. *C. parvum* oocysts in fecal pellets of LS-BuOH extract-treated mice were found to be deformed in shape, lacking inner structure, indicating that *L. spinosa* butanol extract might affect the count and infectivity of the oocysts. A similar result was recorded by Abdel Megeed [59] in a treatment trial using garlic extract against *C. parvum* in pre-weaned buffalo calves. Present results showed that *C. parvum* oocysts and developmental stages were the lowest in mice intestine of both LS-BuOH extract groups (200 mg/Kg and 100 mg/Kg; *P* < 0.05) and this might be due to the reduced severity of infection and lower parasite loads in feces in response to treatment.

Regarding humoral immune response, specific *C. parvum* antibodies IgG elevated significantly in the infected and treated mice (*P < 0.05*) at all day’s post-treatment compared to the infected-untreated ones. Current findings are in accordance with other studies suggesting a significant correlation between IgG response and the intensity of infection [56,63–65]. This might be due to the release of specific antibodies during *C. parvum* infection and could be considered as a marker for cryptosporidiosis [65]. Also, the locally prepared CNBr-activated Sepharose-4B affinity purified antigen was utilized in the indirect ELISA as it had exhibited a high diagnostic potency compared to the crude antigen [8]. IgG levels were highest in the LS-BuOH extract treated group (200 mg/Kg) followed by the mice group treated with LS-BuOH extract (100 mg/Kg) compared to other treated groups. This significant effect might be due to the potential protective and immunomodulatory activity of phenolic compounds present in *L. spinosa,* especially butanol extract [18].

Modulation of the host immune response may also be relevant for selection toward commensalism, since it may prevent deleterious effects to the host resulting from the exacerbated immune response [66]. Concerning cellular immune response, levels of mice serum cytokines; IFN-γ, IL-15 and TNF-α were significantly higher (*P* < 0.001) after 10 and 21 days of infection as compared to healthy mice. These results coincide with those of Aboelsoued et al., 2022 and Ahmad et al., 2013 [56,67], confirming that IFN-γ and IL-15 levels are important for early *C. parvum* control [68–70]. TNF-α was found to play an important role in inflammation and can be involved in protective immunity against intracellular parasites [71,72] as it regulates the growth and differentiation of a variety of cell types [73]. Robinson and his co-workers [74] assumed that TNF-α increased in cryptosporidiosis pathogenesis as it was linked with Lamnia propria histological inflammation in porcine cryptosporidiosis. Regarding IFN-γ and IL-15 levels, mice of the LS-BuOH extract treated group (200 mg/Kg) reached the maximum levels of these cytokines after 10 dPI compared to other infected groups, which then decreased towards a healthy state after 21 dPI (in which, very few oocysts appeared in fecal pellets of this mice group over the week). This might be due to reduced oocyst shedding and lower parasite loads due to the treatment, especially in the LS-BuOH extract group (200 mg/Kg). These findings agree with the association between IFN-γ and the prevention of oocyst shedding [75] and between IL-15 and control of oocyst shedding and elimination of intracellular protozoans from the intestines by activation of an NK cell-mediated pathway [76], as IL-15 is inversely correlated with *Cryptosporidium* burden [77]. In contrast, TNF-α decreased in infected mice treated with *L. spinosa* extracts with the high concentration of LS-BuOH extract at 10 and 21 dPI showcasing superiority, supportive of a role for TNF-α in inflammation which is shown to be minimal in this group after 21 dPI.

Regarding liver enzymes, a significant elevation (*P* < 0.001) in ALT, AST, and ALP in sera of *C. parvum* experimentally infected-untreated mice was detected compared to healthy mice. These findings agreed with Aboelsoued et a1. [26] and Elmahallawy et al. [78] who recorded an elevation in ALT, AST and ALP in *Cryptosporidium* experimentally infected mice. The present results indicated that cryptosporidiosis might be a major factor for severe liver injuries [79,80], hepatocellular damage mediated by *C. parvum* infection in mice [78], and confirmed the extra-intestinal effects of cryptosporidiosis [81]. This serum elevation of ALT could indicate the cell membrane injury while AST could signalize the hepatic tissue mitochondrial damage [82] and ALP could refer to hepatic cellular damage and hepatobiliary disease [83]. After 21 dPI, liver enzyme levels were significantly (*P < *0.001) lower in mice treated with Nitazoxanide or *L. spinosa* extracts, and the best effect was observed in the LS-BuOH extract (200 mg/Kg) treated group where ALT, AST, and ALP were restored towards control healthy levels indicating the strong anti-cryptosporidial effect of LS-BuOH extract against cryptosporidiosis. This effect might be supported by previous reports in *Launaea* species which were traditionally used for the treatment of liver oxidative stress [18,84]. The determination of enzymatic antioxidant activities such as GSH–Px and catalase helps assess oxidative stress [85]. In the current study, the measured GSH–Px and catalase serum activities indicated the oxidative stress in *C. parvum* infected mice suggesting its exaggeration in response to infection [86]. These enzymes were significantly (*P < *0.001) elevated in mice treated with Nitazoxanide or *L. spinosa* extracts with the LS-BuOH extract (200 mg/Kg) treated group showing superiority as their levels enhanced the healthy normal status, suggesting that this extract of *L. spinosa* improved mice health. This could be supported by teamwork of Abdallah [18] results that the major phenolics in *L*. *spinosa* showed a significant cytoprotective effect against oxidative stress which maintains the normal redox status of the cell.

In the present study, intestinal sections of *C. parvum*-infected mice showed many alterations, including lymphoplasmacytic cells infiltration, sloughing and necrosis of villi, congestion of blood vessels, and edema with the presence of *C. parvum* in the intestinal epithelium and crypt of Lieberkühn that suffered from degenerative changes. These observations fall in agreement with previous studies showing the alterations of mice intestinal tissue in response to cryptosporidiosis [26,67,78,87,88]. The reason for this may be the impaired intestinal absorption and barrier function [89], increased paracellular permeability [90], induced innate inflammatory responses, and alteration in the tight junctions between epithelial cells [91]. These pathological alterations might be due to intestinal tissue damage with host cell death or apoptosis in response to this intracellular pathogen [92]. As a result, caspases are activated inducing apoptosis to limit the spreading of infection [93]. In the current study, there was a strong positive expression of cleaved caspase-3 as a marker of infection-induced apoptosis in the intestinal sections of the *C. parvum* infected-untreated mice group. These results matched with Aboelsoued et al. [35], revealing that Caspase-3 was highly expressed in the intestine of *C. parvum*-infected mice, as well as results from Sasahara [94] and Buret [95], suggesting that this epithelial apoptosis could be integral for *C. parvum* pathogenicity and could promote host cell apoptosis. Meanwhile, mice treated with Nitazoxanide and *L. spinosa* extracts showed enhancement of histological characteristics of the intestinal tissue and caspase-3 expression with a marked amelioration in both doses of LS-BuOH extract (100 and 200 mg/Kg). Restoration of the histopathological alterations in intestinal architectures and caspase-3 expression after treatment with *L. spinosa* extracts was significant and was matched with lower mucosal burden and other immunological, biochemical, and inflammatory markers.

With regards to natural product classes reported to exhibit anti-parasitic effects, saponins were found to play a significant role in the *in vitro* inhibition of *C. parvum* oocyst growth [96]. EL-Shewehy et al [97] reported that extracts of *Zingiber officinale*, *Allium sativum*, and *Punica granatum* exert significant anti-cryptosporidium potencies due to the high contents of saponins along with flavonoids and phenolics. According to reports, saponins’ anti-cryptosporidium mechanism of action involves disrupting the protozoal membrane, deactivating enzymes, and depriving cells of materials and metal ions that are essential to cell metabolism [98,99]. El-Sayed and Fathy additionally found that saponins significantly contributed to anti-*Cryptosporidium* impacts through interference with lectin receptors. The potential of *Allium cepa* extract to significantly lower the number of *C. parvum* oocysts has also been associated with flavonoids and sulfated substances [100]. The current chemical profiling of the three extracts of *L. spinosa*, LS-EtOAc, LS-EtOH and LS-BuOH, revealed an abundance of triterpenoid saponins, phenolic acids, and flavonoid conjugates. All these compounds were demonstrated to exhibit strong antioxidant and anti-inflammatory actions [101]. These phenolic and flavonoid components are exogenous antioxidant mediators that function by blocking ion channels, nitric oxide synthase, and xanthine oxide synthase [102], in addition to showcasing antioxidant effects. For example, it was shown that apigenin, luteolin, and their derivatives, as strong antioxidant components, decreased the expression of caspase-3 and other indicators of oxidative stress, including glutathione peroxidase, malondialdehyde, and superoxide dismutase [103]. Phenolics and flavonoids in parsley and dill plants decreased Reactive oxygen species (ROS) levels. Additionally, they strengthen the antioxidant enzyme glutathione-S-transferase (GST) [104]. Partial Least Square correlation results confirmed the significant roles of the triterpenoid saponins, and were in full agreement with previous studies confirming their antioxidant, anti-inflammatory, and antiviral effects [105,106]. Triterpenoid saponins disrupt oocyst formation and prevent parasite sporulation. Sulfated triterpenoidal saponins have garnered attention for their broad spectrum of biological activities, including their antiparasitic potential [105,106]. This property is crucial when considering *Cryptosporidium* treatment, as the parasite’s life cycle involves stages, such as oocysts and sporozoites, that are protected by robust membrane structures. Saponins, particularly those that are sulfated, have demonstrated the ability to disrupt cell membranes, leading to parasite lysis and death, thus reducing infection severity.

While previous studies have reported the antiparasitic potential of plant-derived saponins, sulfated triterpenoidal saponins may offer a distinctive advantage due to their enhanced water solubility and bioavailability. This can potentially improve their interaction with *Cryptosporidium* at different life stages, especially during the intestinal sporozoite invasion. Additionally, their ability to modulate host immune responses, stimulate mucosal immunity, and act as adjuvants could provide a dual mechanism of action—both directly attacking the parasite while also enhancing the host’s defenses. To further differentiate this study, it would be essential to investigate whether the sulfation pattern influences saponin activity against *Cryptosporidium*, which could highlight a unique therapeutic mechanism compared to other non-sulfated saponins documented in existing literature.

## Conclusion

Annually, gastrointestinal cases and waterborne disease outbreaks are primarily brought about by *C. parvum* throughout the globe*. L. spinosa* has traditionally been employed to treat several diseases including gastrointestinal diseases. Fecal *C. parvum* oocyst count and mucosal load were decreased by *L. spinosa* extracts. The levels of IgG, IFN-γ, and IL-15 along with TNF-α were all restored to a healthy state. Additionally, upon extract administration, levels of liver enzymes were reduced while GSH-Px and catalase rose. The histo- and immunohistopathological findings, which demonstrated that *L. spinosa* extracts reduced cleaved caspase-3, damage score, and degenerative alterations expressions, corroborate the above findings. Herein, the anti-cryptosporidial potency of the three extracts of *L. spinosa* was evaluated and found to be most effective in the following order: LS-BuOH>  LS-EtOH>  LS-EtOAc. This study reports the presence of sulfated triterpenoid saponins in *Launaea* genus for the first time in the literature, alongside several other new metabolites. Biological effects were shown to be strongly related to LS-BuOH extract (R2: 0.9) according to PLS regression analysis, with triterpenoid saponins and flavonoid glycosides primarily responsible for the anti-parasitic effect. The outcomes of the present study suggest that *L. spinosa* extracts could be an innovative therapy for *C. parvum* management yet to be tested in clinical trials.

## Supporting information

S1 File Fig. S1 ESI-MS/MS spectrum of peak **10**, **17**, **18**, and **21** in the negative ion mode. Fig. S2 ESI-MS/MS spectrum of peak **26**, **37**, and **42** in the negative ion mode. Fig. S3 ESI-MS/MS spectrum of peak **8** and **12** in the negative ion mode. Fig. S4 ESI-MS/MS spectrum of peak **2** in the negative ion mode. Fig. S5 ESI-MS/MS spectrum of peak **34** in the negative ion mode. Fig. S6 ESI-MS/MS spectrum of peak **33** in the negative ion mode. Fig. S7 ESI-MS/MS spectrum of peak **56** in the negative ion mode. Fig. S8 ESI-MS/MS spectrum of peak **47** in the negative ion mode. Fig. S9 ESI-MS/MS spectrum of peak **68** in the negative ion mode. Fig. S10 ESI-MS/MS spectrum of peak **67** in the negative ion mode.
(DOCX)

S1 Raw data Launea Crypto *Raw data.xlsx* The raw data of (i) Reduction % in C
*. parvum* oocysts’ shedding, (ii) Serum IgG level, (iii) Serum INF-γ, IL-15 and TNF-α levels, (iv) Serum liver function parameters (ALT, AST and ALP), and (v) Antioxidant activity parameters (GSH-Px and Catalase).
(XLSX)
