# Supplementary material for: Anticryptosporidial action mechanisms of Launaea spinosa extracts in Cryptosporidium parvum experimentally infected mice in relation to its UHPLC-MS metabolite profile and biochemometric tools
Source: PLoS One. 2025 Mar 3;20(3):e0317497. doi: 10.1371/journal.pone.0317497 (PMC11875388; doi:10.1371/journal.pone.0317497)
Supplement: S1 File — Fig. S1 ESI-MS/MS spectrum of peak 10, 17, 18, and 21 in the negative ion mode. Fig. S2 ESI-MS/MS spectrum of peak 26, 37, and 42 in the negative ion mode. Fig. S3 ESI-MS/MS spectrum of peak 8 and 12 in the negative ion mode. Fig. S4 ESI-MS/MS spectrum of peak 2 in the negative ion mode. Fig. S5 ESI-MS/MS spectrum of peak 34 in the negative ion mode. Fig. S6 ESI-MS/MS spectrum of peak 33 in the negative ion mode. Fig. S7 ESI-MS/MS spectrum of peak 56 in the negative ion mode. Fig. S8 ESI-MS/MS spectrum of peak 47 in the negative ion mode. Fig. S9 ESI-MS/MS spectrum of peak 68 in the negative ion mode. Fig. S10 ESI-MS/MS spectrum of peak 67 in the negative ion mode. (DOCX) [file pone.0317497.s001.docx]

**Supporting information**

**Fig. S1** ESI-MS/MS spectrum of peak **10**, **17**, **18**, and **21** in the negative ion mode.

**Fig. S2** ESI-MS/MS spectrum of peak **26**, **37**, and **42** in the negative ion mode.

**Fig. S3** ESI-MS/MS spectrum of peak **8** and **12** in the negative ion mode.

**Fig. S4** ESI-MS/MS spectrum of peak **2** in the negative ion mode.

**Fig. S5** ESI-MS/MS spectrum of peak **34** in the negative ion mode.

**Fig. S6** ESI-MS/MS spectrum of peak **33** in the negative ion mode.

**Fig. S7** ESI-MS/MS spectrum of peak **56** in the negative ion mode.

**Fig. S8** ESI-MS/MS spectrum of peak **47** in the negative ion mode.

**Fig. S9** ESI-MS/MS spectrum of peak **68** in the negative ion mode.

**Fig. S10** ESI-MS/MS spectrum of peak **67** in the negative ion mode.
